# Supplementary material for: Short-term and long-term revision rates after lumbar spine discectomy versus laminectomy: a population-based cohort study
Source: BMJ Open. 2018 Jul 17;8(7):e021028. doi: 10.1136/bmjopen-2017-021028 (PMC6059274; doi:10.1136/bmjopen-2017-021028)
Supplement: Supplementary data [file bmjopen-2017-021028supp001.pdf]

Appendix . ICD-9-CM codes and the corresponding diseases or procedures

Disease or procedures	Corresponding ICD-9-CM codes
Lumbar discectomy	83024C
laminectomy	83002C, 83003C
Spinal fusion	83043B, 83044B, 83045B, 83046B<br>64221B, 64222B, 64224B,64225B, 64226B
Spine fracture	64160B
Ankylosing spondylitis	720; 720.0
Systemic lupus erythematous (SLE)	710.0
Rheumatoid arthritis (RA)	714.XX
Cancers	140.xx-208.xx
Spinal tumor cases	192.2; 192.3; 198.3; 225.3; 225.4; 237.5
Cervical disease	721.x (x =0,1,5,6,7), 722.0, 722.4,<br>722.71, 722.81, 722.91,<br>723.x (x=0 ~ 9)<br>344.xx (xx=00, 01,02,03,04,09)<br>344.1, 344.2, 344.4x (x=0,1,2)<br>805.xx (xx= 00 ~08; 10 ~18)<br>806.xx (xx= 00 ~09; 10 ~19)<br>952.xx (xx=00 ~09)
thoracic disease	721.2, 721.41<br>722.xx (xx=11, 51, 72, 82, 92)<br>724.01<br>805.2, 805.3,<br>806.xx (xx= 20 ~29; 30 ~39),<br>952.xx (xx=10 ~19)
congenital anomaly of spine	756.xx (xx=13,14,15,19),<br>756.4

Tuberculosis of spine (TB)	015.xx (xx= 00~06)
spine infection	711. xx (xx= 08,48,58,68,88,98)<br>730.xx (xx= 08,18,28,38,88,98)
Incidental durotomy	998.2
Post-operative hemorrhage	998.1x (xx= 1,2,3)
Post-operative spine infection	998.3; 998.6<br>998.xx (xx= 51,59,83)<br>711. xx (xx= 08,48,58,68,88,98)<br>730.xx (xx= 08,18,28,38,88,98)
Postlaminectomy syndrome; lumbar region	722.83; 722.80
Lumar disc problem	722.x (x=2, 6,<br>722.xx (xx=10, 52, 70, 73, 90.93)
Acquired spondylolishtesis	738.4; 738.5
Lumbar spinal stenosis	724.02; 724.09
Lumbosacral spondylosis	721.3;<br>721.xx (xx= 42, 90, 91)<br>722.32; 722.39

Footnotes: ICD-9-CM, International Classification of Diseases, 9th Revision, Clinical Modification;
