# Supplementary material for: Short-term and long-term revision rates after lumbar spine discectomy versus laminectomy: a population-based cohort study
Source: BMJ Open. 2018 Jul 17;8(7):e021028. doi: 10.1136/bmjopen-2017-021028 (PMC6059274; doi:10.1136/bmjopen-2017-021028)
Supplement: Supplementary data [file bmjopen-2017-021028supp002.pdf]

TABLE S1.1 The Cause of revision lumbar spine surgery (Unmatched data)

	Discectomy N=4424	Laminectomy N=5228
Incidental durotomy	3(0.07)	1(0.02)
Post-operative hemorrhage	6(0.14)	11(0.21)
Post-operative spine infection	59(1.33)	108(2.07)
Postlaminectomy syndrome; lumbar region	322(7.28)	543(10.39)
Lumar disc problem	2523(57.03)	1409(26.95)
Acquired spondylolishtesis	386(8.73)	753(14.4)
Lumbar spinal stenosis	605(13.68)	1142(21.84)
Lumbosacral spondylosis	520(11.75)	1261(24.12)

TABLE S1.2 The Cause of revision lumbar spine surgery (matched data)

	Discectomy N=1124	Laminectomy N=977
Incidental durotomy	2(0.18)	1(0.1)
Post-operative hemorrhage	2(0.18)	3(0.31)
Post-operative spine infection	20(1.78)	18(1.84)
Postlaminectomy syndrome; lumbar region	78(6.94)	86(8.8)
Lumar disc problem	763(67.88)	442(45.24)
Acquired spondylolishtesis	53(4.72)	74(7.57)
Lumbar spinal stenosis	93(8.27)	131(13.41)
Lumbosacral spondylosis	113(10.05)	222(22.72)
