# Supplementary material for: Short-term and long-term revision rates after lumbar spine discectomy versus laminectomy: a population-based cohort study
Source: BMJ Open. 2018 Jul 17;8(7):e021028. doi: 10.1136/bmjopen-2017-021028 (PMC6059274; doi:10.1136/bmjopen-2017-021028)
Supplement: Author's manuscript [file bmjopen-2017-021028.draft_revisions.pdf]

BMJ Open is committed to open peer review. As part of this commitment we make the peer review history of every article we publish publicly available.

When an article is published we post the peer reviewers' comments and the authors' responses online. We also post the versions of the paper that were used during peer review. These are the versions that the peer review comments apply to.

The versions of the paper that follow are the versions that were submitted during the peer review process. They are not the versions of record or the final published versions. They should not be cited or distributed as the published version of this manuscript.

BMJ Open is an open access journal and the full, final, typeset and author-corrected version of record of the manuscript is available on our site with no access controls, subscription charges or pay-per-view fees (http://bmjopen.bmj.com).

If you have any questions on BMJ Open's open peer review process please email info.bmjopen@bmj.com

BMJ Open

Long-term Revision Rate after Lumbar Spine Disectomy or Laminectomy: A Population-Based Cohort Study

Journal:	*BMJ Open*
Manuscript ID	bmjopen-2017-021028
Article Type:	Research
Date Submitted by the Author:	21-Dec-2017

Complete List of Authors:	kao, Feng-Chen; E-Da Hospital<br>Hsu, Yao-chun; Fu-Jen Catholic University Hospital, Gastroenterology<br>Wang, Chang-Bi ; China Medical University, Clinical Medicine<br>Tu, Yuan-Kun; E-Da Hospital, Orthopedics<br>Liu, Pao-Hsin ; I-Shou University College of Medicine, Biomedical Engineering

Keywords:	disectomy, laminectomy, reoperation, revision rate

SCHOLARONE™
Manuscripts

For peer review only

Long-term Revision Rate after Lumbar Spine Disectomy or Laminectomy: A Population-Based Cohort Study

Feng-Chen Kao[1,2], MD, PhD; Yao-Chun Hsu[3-6], MD, PhD; Chang-Bi Wang PhD[7], Yuan-Kun Tu[1,2], MD, PhD; Pao-Hsin Liu [8]PhD

[1]Department of Orthopedics, E-Da Hospital, Kaohsiung, Taiwan;

[2]School of Medicine for International Students, I-Shou University, Kaohsiung, Taiwan;

[3] School of Medicine, [2]Big Data Research Center, Fu-Jen Catholic University, New Taipei, Taiwan;

[4]Division of Gastroenterology, Fu-Jen Catholic University Hospital, New Taipei, Taiwan

[5]Graduate Institute of Clinical Medicine, China Medical University, Taichung, Taiwan

[6]Division of Gastroenterology and Hepatology, E-Da Hospital, Kaohsiung, Taiwan

[7] Graduate Institute of Public Health, China Medical University, Taichung, Taiwan

[8]Department of Biomedical Engineering, I-Shou University , Kaohsiung, Taiwan

Correspondence to ***Yao-Chun Hsu***

Dr. ***Yao-Chun Hsu***, MD, PhD; Department of Division of Gastroenterology, Fu-Jen Catholic University Hospital; No.69, Guizi Rd., Taishan Dist., New Taipei City 24352, Taiwan Tel: +886-7-615-1100; ext. 5903; Fax: +886-7-6150940;

Email: holdenhsu@ntu.edu.tw

ABSTRACT

For peer review only - http://bmjopen.bmj.com/site/about/guidelines.xhtml

Background/Objective: The natural history of a degenerative spine dictates a high incidence of surgical revision after lumbar spine disectomy or laminectomy. However, the long-term revision rates between the two procedures remained unclear.

Design: Population-based cohort study

Setting: Data from the Taiwan National Health Insurance Research Database

Patients or Participants: We identified and enrolled patients who underwent lumbar disectomy or laminectomy for the first time between January 1, 1997, and December 31, 2007. All patients were followed up for 5 years or until death.

Results: The revision rate within 3 months of the index surgery was significantly higher in patients who underwent disectomy (2.75%) than in those undergoing laminectomy (1.18%; $p < 0.0001$). The difference persisted within one year of the index operation (3.38% vs. 2.57%). One year afterward, the revision rates were similar between disectomy (9.75%) and laminectomy (9.69%). The final spinal fusion surgery rates were also similar in both groups (11.25% vs. 12.08%).

Conclusion: The revision rate after lumbar disectomy was higher than that after laminectomy within 1 year of the index operation. However, the two procedures were not different in long-term revision rates and the need of final spinal fusion surgery.

Article summary

1. The natural history of the degenerative spine is expected to lead prevalence of revision surgery.
2. Our study is a population-based cohort study include whole Taiwan's people by Analysing of the Taiwan National Health Insurance Research Database
3. The reoperation rate after lumbar disectomy is higher than that after lumbar laminectomy withing one year after first time lumbar surgery.
4. Beyond one year after first time lumbar surgery, the reoperation rate and final

For peer review only - http://bmjopen.bmj.com/site/about/guidelines.xhtml

lumbar spinal fusion rate are similar in disectomy and laminectomy groups.

5. We have limitations: First, laboratory, radiographic and pathological data were unavailable in the NHIRD. Second, the physical condition of these patients could not be evaluated, which might lead to a healthy patient bias.

Keywords: disectomy, laminectomy, reoperation, revision rate

For peer review only

For peer review only - http://bmjopen.bmj.com/site/about/guidelines.xhtml

INTRODUCTION

The natural history of the degenerative spine dictates an inevitable occurrence of primary disc herniation and lumbar spinal stenosis, and most of these patients are treated through surgical interventions.[1 2] Lumbar disc herniation is a common manifestation of degenerative lumbar disc disease.[3-5] It occurs early in the degenerative cascade and represents the tensile failure of the annulus to contain the gel-like nuclear portion of the disc. Treatment for lumbar herniated discs can be challenging, although nonoperative treatment is effective in the majority of cases.[6 7] Other studies have indicated that surgery provides superior results, especially for short-term pain relief.[3 8]

Lumbar spinal stenosis is a progressive and dynamic disease, which is best considered on a continuum of pathological changes occurring in the spinal column during aging. The incidence of lumbar spinal stenosis increases during the fifth decade of life and ranges from 1.7% to 8% in the general population.[9] The principal aims of surgery are focused on individuals' pathological anatomy and involve relieving the neurologic compression, which is likely more complex than simple compression.[10]

Accordingly, an expected prevalence of revision surgery is noted.[11 12] Revision surgery is always a challenge for the spinal surgeon, and particular care is necessary in identifying appropriate clinical situations for additional surgery. The surgeon should be attuned to these suitable clinical circumstances and be technically qualified to address the unique anatomic and pathologic milieus posed by repeat surgery. The incidence of revision surgery after lumbar surgical discetomy varies widely, from 0% to approximately 15%.[1] Frymoyer[13] reported the incidence of postdisectomy

For peer review only - http://bmjopen.bmj.com/site/about/guidelines.xhtml

instability requiring further spinal fusion surgery as up to 6.5%. Reports specifically addressing revision surgery for lumbar spinal stenosis are relatively few, although Malter and colleagues[12] reported that the 5-year reoperation rate for patients with spinal stenosis was up to 12%.

To clarify whether the spinal reoperation rates differ after lumbar disectomy and laminectomy for lumbar spinal stenosis, we performed a population-based retrospective study of the 5-year follow-up data of patients from the Taiwan National Health Insurance Research Database.

For peer review only

MATERIALS AND METHODS

Data Source

We examined data from the Taiwan National Health Research Institute Database (NHIRD), which are released by the Taiwan National Health Research Institute (NHRI) for public use. The NHRI covers the medical claims of 22.9 million residents, which accounts for >99% of the total population of Taiwan. The NHIRD includes the claims data from 1997 to 2013. The Department of Health and the National Health Insurance (NHI) Bureau of Taiwan ensure the completeness and accuracy of the NHIRD. This study was exempted from an ethics review because the medical records released by the insurance authority are encrypted secondary data and could be used for research purposes.

This retrospective population-based cohort study used the data from the Longitudinal NHIRD. Until the end of 2013, all sampled individuals were followed up for outcome identification by using the International Classification of Disease, Ninth Revision, Clinical Modification (ICD-9-CM) codes. This study was approved by the institutional review board of our hospital (EMRP-104-04) and the Taiwan NHRI (NHIRD-103-116). This study was exempted from a full review by the institutional review board of E-Da Hospital.

Definition of Study Cohorts and Outcomes

We included patients from the NHIRD, who underwent lumbar disectomy or laminectomy for the first time between January 1, 1997, and December 31, 2007, in our study cohort. Those who received their first lumbar disectomy or laminectomy after 2007 were excluded because dynamic stabilization systems, such as the "Wallis"

For peer review only - http://bmjopen.bmj.com/site/about/guidelines.xhtml

system,[14] were marketed in Taiwan after 2007. We also excluded individuals who were continuously exposed to oral or injected forms of systemic corticosteroids for a minimum of 6 months, and those who had diseases such as ankylosing spondylitis, systemic lupus erythematous, rheumatoid arthritis, malignant cancers, spinal tumors, congenital spinal anomalies, spinal tuberculosis, spinal infections, spinal fractures, cervical spinal disease, and thoracic spinal disease. The corresponding ICD-9-CM codes are listed in Appendix 1.

We divided the study cohort into disectomy and laminectomy groups. The date of discharge from the hospital after the first lumbar discectomy or laminectomy was considered the index date. Revision lumbar spine surgery was defined as a second lumbar spine surgery performed after the index date and comprised the following types: lumbar spine disectomy, lumbar spine laminectomy (including laminotomy), and lumbar spinal fusion surgery (with or without instrumentation). The revision rates were evaluated and compared between the two surgical groups. The date of discharge from the hospital after first time lumbar disectomy or laminectomy was assigned as the index day. The two groups were also propensity score–matched at a ratio of 1:1, according to the baseline characteristics of those patients. (however, we assessed both unmatched and matched data in this study). Comorbidities existing prior to the index date were classified according to the Charlson score.[15] The mortality rates after the index date were also calculated in both groups. The revision rates were compared including mortality rates to eliminate the influence of death on the likelihood of revision surgery. We also calculated and compared the rates for final revision spinal fusion surgeries between those two groups. All the patients were followed up until death, withdrawal from the NHI program, or December 31, 2012.

Statistical Analysis

For peer review only - http://bmjopen.bmj.com/site/about/guidelines.xhtml

We used the Pearson chi-square test, Fisher exact test and Yates continuity correction, and t test to compare quantitative data. Data were evaluated using the log-rank test and univariate and multivariate Cox regression analyses. All p values <0.05 were considered significant. All statistical tests and hazard ratio (HR) calculations were performed using Statistical Analysis Software, Version 9.4 (SAS Institute, Cary, NC, USA).

For peer review only

For peer review only - http://bmjopen.bmj.com/site/about/guidelines.xhtml

RESULTS

Baseline Characteristics of the Patients

We included 66,754 patients (31,964 female and 34,790 male) in this study cohort. The unmatched and matched baseline characteristics, as well as the comorbidities, of all patients are listed in Tables 1.1 and 1.2. After propensity score matching, 8024 patients were included in both groups.

Total Spinal Revision Rates After the First Spinal Surgeries

Significant differences were observed in the total revision spinal surgery rates between patients who received lumbar disectomy and those who received lumbar laminectomy as the first surgery. In the unmatched data, the revision spinal surgery rates in the disectomy and laminectomy groups were 15.88% and 13.44%, respectively ($p < 0.0001$). In the matched data, the rates were 14.01% and 12.18%, respectively ($p < 0.001$). The cumulated incidence of total revision spinal surgery is shown in Fig. 1.

Rates for Revision Surgeries Performed Within 3 Months of the First Spinal Surgeries

The rates for revision spinal surgeries performed within 3 months of the first spinal surgeries significantly differed between patients who received lumbar disectomy and those who received lumbar laminectomy ($p < 0.0001$). In the unmatched data, the revision spinal surgery rates in the disectomy and laminectomy groups were 2.75% and 1.18%, respectively. In the matched data, the rates were 2.59% and 1.53%, respectively.

For peer review only

For peer review only - http://bmjopen.bmj.com/site/about/guidelines.xhtml

Rates for Revision Surgeries Performed Between 3 Months and 1 Year After the First Spinal Surgeries

The rates for revision spinal surgeries performed between 3 months and 1 year after the first spinal surgeries also significantly differed between patients who received lumbar disectomy and those who received lumbar laminectomy. In the unmatched data, the revision spinal surgery rates in the disectomy and laminectomy groups were 3.38% and 2.57%, respectively ($p < 0.0001$). In the matched data, the rates were 3.00% and 2.36%, respectively ($p < 0.05$).

Rates for Revision Surgeries Performed More Than 1 Year After the First Spinal Surgeries

The rates for revision spinal surgeries performed more than 1 year after the first spinal surgeries did not significantly differ between patients who received lumbar disectomy and those who received lumbar laminectomy. In the unmatched data, the revision spinal surgery rates in the disectomy and laminectomy groups were 9.75% and 9.69%, respectively. In the matched data, the rates were 8.41% and 8.29%, respectively.

Differences in Multivariate-Adjusted Total Revision Spinal Surgery Rates Between the Disectomy and Laminectomy Groups

The multivariate-adjusted Cox proportional hazards model revealed independent differences in the unmatched and matched data (adjusted HR, 0.81 and 0.86, respectively; 95% confidence interval [CI], 0.78–0.85 and 0.79–0.94, respectively; Table 2) between the disectomy and laminectomy groups.

For peer review only - http://bmjopen.bmj.com/site/about/guidelines.xhtml

Rates for Final Spinal Fusion Surgeries Performed After the First Spinal Surgeries

No significant differences were observed in the rates for final spinal fusion surgeries performed after the first spinal surgeries between patients who received lumbar disectomy and those who received lumbar laminectomy. In the unmatched data, the final spinal fusion surgery rates in the disectomy and laminectomy groups were 11.25% and 12.08%, respectively. In the matched data, the rates were 9.77% and 10.44%, respectively. The cumulated incidence of final spinal fusion surgeries performed after the first spinal surgeries is shown in Fig. 2.

Differences in Multivariate-Adjusted Final Spinal Fusion Surgery Rates Performed After the First Spinal Surgeries Between the Disectomy and Laminectomy Groups

The multivariate-adjusted Cox proportional hazards model revealed no differences in the unmatched data between the disectomy and laminectomy groups (adjusted HR, 1.05; 95% CI, 1.00–1.10; Table 3). However, the multivariate-adjusted Cox proportional hazards model revealed independent differences in the matched data between the disectomy and laminectomy groups (adjusted HR, 1.11; 95% CI, 1.01–1.22).

For peer review only - http://bmjopen.bmj.com/site/about/guidelines.xhtml

DISCUSSION

Lumbar disc herniation is one of the most common disorders of the lumbar spine.[16] In 1934, Mixter and Barr[17] identified the link between sciatica and herniation of a lumbar disc; since then, disectomy through a limited laminotomy remains the most common surgical management for the prolapse of a lumbar disc, following the failure of conservative management.[18] The efficacy of lumbar discectomy for the treatment of lumbar disc herniation has been demonstrated[19 20]; however, unsatisfactory outcomes after lumbar disectomy have also been reported in approximately 5%–20% of cases.[21-24] The SPORT trial reported that in patients with lumbar disc herniation, the rates of reoperation within 4 and 8 years of the index procedure were as high as 9% and 13%, respectively.[19] The most common cause of ongoing disability after lumbar discectomy is recurrent lumbar disc herniation, which occurs in 5%–15% of patients (notably, this incidence rate increases over time).[21 23 25-28] In our series, the rates for revision spinal surgeries performed within 3 months and 1 year of lumbar disectomy were 2.75% and 3.38%, respectively; the rates for revision surgery performed after 1 year and the total revision surgery rates were 9.75% and 15.88%, respectively.

Lumbar stenosis occurs due to spondylotic changes in the facet joints, instability, or a congenitally small canal.[29] Laminectomy remains the gold standard for treating spinal stenosis in the absence of spinal instability.[29] Despite adequate lumbar decompression, substantial back and leg pain occurs in up to 10%–15% of patients postoperatively.[30] Historically, lumbar laminectomy has a high rate of failure, and the incidence of recurrent back pain can reach up to 47%.[31 32] Currently, no reports exist on the reoperation rates after lumbar laminectomy without spinal fusion surgery. In our series, the rates for revision spinal surgery performed within 3 months and 1

For peer review only - http://bmjopen.bmj.com/site/about/guidelines.xhtml

year of lumbar laminectomy were 1.18% and 2.57%, respectively; the rates for revision surgery performed after 1 year and the total revision surgery rates were 9.69% and 13.44%, respectively.

The degree of contribution of spinal structures providing spinal stability are as follows: facet capsule, 39%; disc and annulus, 29%; supraspinous and intraspinous ligaments, 19%; and ligamentum flavum, 13%.[33] Interventions at the hemilamina and ligamentum flavum can change both the load-bearing and kinematic characteristics of the spine and subsequently lead to spinal segment hypermobility and accelerated bone degeneration.[34 35] Even microdiscectomy can increase the risk of single-level instability,[36] and extensive laminectomy can potentate spinal instability.[37 38] Lai[39] reported that sacrificing either the supraspinous ligament or the tendon insertion points on the spinous processes can lead to an accelerated development of adjacent instability. The incidence of adjacent instability increases with the amount of destructed lamina, and the amount of destructed posterior spinal complexes is substantially greater in lumbar laminectomy than in lumbar disectomy. Theoretically, more spinal instability would occur after lumbar laminectomy than after lumbar disectomy; hence, the reoperation rate should be higher after lumbar laminectomy.

However, our study revealed independent differences in the reoperation rates according to the unmatched and matched data (adjusted HR, 0.81 and 0.86; 95% CI, 0.78–0.85 and 0.79–0.94; respectively) between the disectomy and laminectomy groups. In the unmatched data, the revision spinal surgery rates in the disectomy and laminectomy groups were 15.88% and 13.44%, respectively ($p < 0.0001$). In the matched data, the revision spinal surgery rates in the disectomy and laminectomy groups were 14.01% and 12.18%, respectively ($p < 0.001$). Compared with the

For peer review only - http://bmjopen.bmj.com/site/about/guidelines.xhtml

laminectomy group, the disectomy group had higher rates of reoperation within 3 months and between 3 months and 1 year after the first surgeries ($p < 0.05$). However, beyond 1 year, the reoperation rates did not significantly differ between the laminectomy and disectomy groups.

Many reasons for reoperation after disectomy have been suggested. Early recurrence may be due to re-herniation, infection, and arachnoiditis; late recurrence may be attributed to foraminal stenosis, a painful disc, epidural fibrosis, iatrogenic segmental instability, progressive facet degeneration, or sacroiliac joint pain.[40-42] The outcomes for natural degeneration of the lumbar spine more than 1 year after the first lumbar spine surgeries were similar in both the disectomy and laminectomy groups.

North et al.[43] reported that the incidence of instability increases from 12.5% after the first revision surgery to 50% after the fourth surgery. Moreover, fusion of the symptomatic spinal segment during revision spinal surgery is related to a successful outcome.[44-47] In our study, no significant differences were observed in the final spinal fusion surgery rates after the first spinal surgeries between patients who received lumbar disectomy (11.25%) and those who received lumbar laminectomy (12.08%).

Our study has some limitations to consider. First, the laboratory, radiographic, and pathological data of the patients were unavailable in the NHIRD. Second, the physical condition of these patients could not be evaluated, which might have led to a healthy patient bias. Nevertheless, this stringent definition would have biased the result toward a null association instead of creating a spurious one. In addition, the potential influence of body weight, cigarette smoking, alcohol drinking, and dietary habits could not be assessed because this information was unavailable in the NHIRD.

For peer review only - http://bmjopen.bmj.com/site/about/guidelines.xhtml

Additionally, because linking the NHIRD with external databases is strictly prohibited for privacy protection, we could not acquire direct information on these factors. However, the NHIRD includes information on all of the residents of Taiwan. A notable strength of our study is that no patients were lost to follow-up, which was particularly due to convenient hospital travel.

In conclusion, the rates for reoperation within 1 year were higher after lumbar disectomy than after lumbar laminectomy. Beyond 1 year after the first lumbar surgery, the reoperation rate and final lumbar spinal fusion surgery rate were similar in the disectomy and laminectomy groups.

ACKNOWLEDGMENTS

This study used data from the National Health Insurance Research Database, which is provided by the Bureau of National Health Insurance, Department of Health, and managed by the National Health Research Institutes. The interpretation and conclusions contained herein do not represent the views of the Bureau of National Health Insurance, Department of Health, or National Health Research Institutes. The authors are grateful to Ms. Tzu-Shan Chen for her efficient assistance.

A. contributor ship statement

construct idea of this study: Hsu and Tu

wrote paper: Kao

data collection and analysis: Wang and Liu

review and revise paper: Kao, Hsu and Liu

For peer review only - http://bmjopen.bmj.com/site/about/guidelines.xhtml

B. competing interests: This study is only considered published in BMJ Open journal.

C. funding: This work was supported by the Center for Database Research, E-DA HEALTHCARE GROUP and E-Da Hospital (EDAHP103048).

D. data sharing statement: This study is based on data from the National Health Insurance Research Database provided by the Bureau of National Health Insurance, Department of Health and managed by National Health Research Institutes. The interpretation and conclusions contained herein do not represent those of Bureau of National Health Insurance, the Department of Health or National Health Research Institutes.

REFERENCES

1. Hoffman RM, Wheeler KJ, Deyo RA. Surgery for herniated lumbar discs: a literature synthesis. Journal of general internal medicine 1993;**8**(9):487-96
2. McCulloch JA. Focus issue on lumbar disc herniation: macro- and microdiscectomy. Spine 1996;**21**(24 Suppl):45s-56s
3. Atlas SJ, Keller RB, Chang Y, et al. Surgical and nonsurgical management of sciatica secondary to a lumbar disc herniation: five-year outcomes from the Maine Lumbar Spine Study. Spine 2001;**26**(10):1179-87
4. DePalma AF, Rothman RH. Surgery of the lumbar spine. Clinical orthopaedics and related research 1969;**63**:162-70
5. Fisher RG, Saunders RL. Lumbar disc protrusion in children. Journal of neurosurgery 1981;**54**(4):480-3 doi: 10.3171/jns.1981.54.4.0480[published Online First: Epub Date]|.
6. Saal JA, Saal JS. Nonoperative treatment of herniated lumbar intervertebral disc with radiculopathy. An outcome study. Spine 1989;**14**(4):431-7
7. Saal JA, Saal JS, Herzog RJ. The natural history of lumbar intervertebral disc extrusions treated nonoperatively. Spine 1990;**15**(7):683-6
8. Weber H. Lumbar disc herniation. A controlled, prospective study with ten years of observation. Spine 1983;**8**(2):131-40
9. Rosen CD, Kahanovitz N, Bernstein R, et al. A retrospective analysis of the efficacy of epidural steroid injections. Clinical orthopaedics and related research 1988(228):270-2
10. Porter RW. Spinal stenosis and neurogenic claudication. Spine 1996;**21**(17):2046-52
11. Keskimaki I, Seitsalo S, Osterman H, et al. Reoperations after lumbar disc surgery: a population-based study of regional and interspecialty variations. Spine 2000;**25**(12):1500-8
12. Malter AD, McNeney B, Loeser JD, et al. 5-year reoperation rates after different types of lumbar spine surgery. Spine 1998;**23**(7):814-20
13. Frymoyer JW. Back pain and sciatica. The New England journal of medicine

For peer review only - http://bmjopen.bmj.com/site/about/guidelines.xhtml

1988;**318**(5):291-300 doi: 10.1056/nejm198802043180506[published Online First: Epub Date]|.

14. Senegas J. Mechanical supplementation by non-rigid fixation in degenerative intervertebral lumbar segments: the Wallis system. European spine journal : official publication of the European Spine Society, the European Spinal Deformity Society, and the European Section of the Cervical Spine Research Society 2002;**11 Suppl 2**:S164-9 doi: 10.1007/s00586-002-0423-9[published Online First: Epub Date]|.
15. Charlson ME, Pompei P, Ales KL, et al. A new method of classifying prognostic comorbidity in longitudinal studies: development and validation. Journal of chronic diseases 1987;**40**(5):373-83
16. Li Z, Tang J, Hou S, et al. Four-year follow-up results of transforaminal lumbar interbody fusion as revision surgery for recurrent lumbar disc herniation after conventional discectomy. Journal of clinical neuroscience : official journal of the Neurosurgical Society of Australasia 2015;**22**(2):331-7 doi: 10.1016/j.jocn.2014.06.098[published Online First: Epub Date]|.
17. MIXTER WJ, BARR JS. Rupture of the Intervertebral Disc with Involvement of the Spinal Canal. New England Journal of Medicine 1934;**211**(5):210-15 doi: 10.1056/nejm193408022110506[published Online First: Epub Date]|.
18. Morgan-Hough CV, Jones PW, Eisenstein SM. Primary and revision lumbar discectomy. A 16-year review from one centre. The Journal of bone and joint surgery British volume 2003;**85**(6):871-4
19. Lurie JD, Tosteson TD, Tosteson AN, et al. Surgical versus nonoperative treatment for lumbar disc herniation: eight-year results for the spine patient outcomes research trial. Spine 2014;**39**(1):3-16 doi: 10.1097/brs.0000000000000088[published Online First: Epub Date]|.
20. Yorimitsu E, Chiba K, Toyama Y, et al. Long-term outcomes of standard discectomy for lumbar disc herniation: a follow-up study of more than 10 years. Spine 2001;**26**(6):652-7
21. Cinotti G, Roysam GS, Eisenstein SM, et al. Ipsilateral recurrent lumbar disc herniation. A prospective, controlled study. The Journal of bone and joint surgery British volume 1998;**80**(5):825-32
22. Cheng J, Wang H, Zheng W, et al. Reoperation after lumbar disc surgery in two hundred and seven patients. International orthopaedics 2013;**37**(8):1511-7 doi: 10.1007/s00264-013-1925-2[published Online First: Epub Date]|.
23. Aizawa T, Ozawa H, Kusakabe T, et al. Reoperation for recurrent lumbar disc herniation: a study over a 20-year period in a Japanese population. Journal of orthopaedic science : official journal of the Japanese Orthopaedic Association 2012;**17**(2):107-13 doi: 10.1007/s00776-011-0184-6[published Online First: Epub Date]|.
24. Carragee EJ, Han MY, Suen PW, et al. Clinical outcomes after lumbar discectomy for sciatica: the effects of fragment type and anular competence. The Journal of bone and joint surgery American volume 2003;**85-a**(1):102-8
25. Swartz KR, Trost GR. Recurrent lumbar disc herniation. Neurosurgical focus 2003;**15**(3):E10
26. Ruetten S, Komp M, Merk H, et al. Recurrent lumbar disc herniation after conventional discectomy: a prospective, randomized study comparing full-endoscopic interlaminar and transforaminal versus microsurgical revision. Journal of spinal disorders & techniques 2009;**22**(2):122-9 doi: 10.1097/BSD.0b013e318175ddb4[published Online First: Epub Date]|.

For peer review only - http://bmjopen.bmj.com/site/about/guidelines.xhtml

27. Ambrossi GL, McGirt MJ, Sciubba DM, et al. Recurrent lumbar disc herniation after single-level lumbar discectomy: incidence and health care cost analysis. Neurosurgery 2009;**65**(3):574-8; discussion 78 doi: 10.1227/01.neu.0000350224.36213.f9[published Online First: Epub Date]|.
28. Miwa S, Yokogawa A, Kobayashi T, et al. Risk factors of recurrent lumbar disk herniation: a single center study and review of the literature. Journal of spinal disorders & techniques 2015;**28**(5):E265-9 doi: 10.1097/BSD.0b013e31828215b3[published Online First: Epub Date]|.
29. Herkowitz H, Garfin, Eismont, Bell & Balderston. *Rothman-Simeone The Spine*. 6 ed, 2011.
30. Booth KC, Bridwell KH, Eisenberg BA, et al. Minimum 5-year results of degenerative spondylolisthesis treated with decompression and instrumented posterior fusion. Spine 1999;**24**(16):1721-7
31. Jackson RK. The long-term effects of wide laminectomy for lumbar disc excision. A review of 130 patients. The Journal of bone and joint surgery British volume 1971;**53**(4):609-16
32. Fritsch EW, Heisel J, Rupp S. The failed back surgery syndrome: reasons, intraoperative findings, and long-term results: a report of 182 operative treatments. Spine 1996;**21**(5):626-33
33. Adams MA, Hutton WC. The mechanical function of the lumbar apophyseal joints. Spine 1983;**8**(3):327-30
34. Kaigle AM, Holm SH, Hansson TH. Experimental instability in the lumbar spine. Spine 1995;**20**(4):421-30
35. Nachemson A. Lumbar spine instability. A critical update and symposium summary. Spine 1985;**10**(3):290-1
36. Schaller B. Failed back surgery syndrome: the role of symptomatic segmental single-level instability after lumbar microdiscectomy. European spine journal : official publication of the European Spine Society, the European Spinal Deformity Society, and the European Section of the Cervical Spine Research Society 2004;**13**(3):193-8 doi: 10.1007/s00586-003-0632-x[published Online First: Epub Date]|.
37. Ebara S, Harada T, Hosono N, et al. Intraoperative measurement of lumbar spinal instability. Spine 1992;**17**(3 Suppl):S44-50
38. Iida Y, Kataoka O, Sho T, et al. Postoperative lumbar spinal instability occurring or progressing secondary to laminectomy. Spine 1990;**15**(11):1186-9
39. Lai PL, Chen LH, Niu CC, et al. Relation between laminectomy and development of adjacent segment instability after lumbar fusion with pedicle fixation. Spine 2004;**29**(22):2527-32; discussion 32
40. Slipman CW, Shin CH, Patel RK, et al. Etiologies of failed back surgery syndrome. Pain medicine (Malden, Mass) 2002;**3**(3):200-14; discussion 14-7 doi: 10.1046/j.1526-4637.2002.02033.x[published Online First: Epub Date]|.
41. Burton CV, Kirkaldy-Willis WH, Yong-Hing K, et al. Causes of failure of surgery on the lumbar spine. Clinical orthopaedics and related research 1981(157):191-9
42. Waguespack A, Schofferman J, Slosar P, et al. Etiology of long-term failures of lumbar spine surgery. Pain medicine (Malden, Mass) 2002;**3**(1):18-22 doi: 10.1046/j.1526-4637.2002.02007.x[published Online First: Epub Date]|.
43. North RB, Campbell JN, James CS, et al. Failed back surgery syndrome: 5-year follow-up in 102 patients undergoing repeated operation. Neurosurgery 1991;**28**(5):685-90; discussion 90-1

For peer review only - http://bmjopen.bmj.com/site/about/guidelines.xhtml

44. Lakkol S, Bhatia C, Taranu R, et al. Efficacy of less invasive posterior lumbar interbody fusion as revision surgery for patients with recurrent symptoms after discectomy. The Journal of bone and joint surgery British volume 2011;**93**(11):1518-23 doi: 10.1302/0301-620x.93b11.27187[published Online First: Epub Date]|.
45. Wong CB, Chen WJ, Chen LH, et al. Clinical outcomes of revision lumbar spinal surgery: 124 patients with a minimum of two years of follow-up. Chang Gung medical journal 2002;**25**(3):175-82
46. Laus M, Alfonso C, Tigani D, et al. Failed back syndrome: a study on 95 patients submitted to reintervention after lumbar nerve root decompression for the treatment of spondylotic lesions. La Chirurgia degli organi di movimento 1994;**79**(1):119-26
47. Duggal N, Mendiondo I, Pares HR, et al. Anterior lumbar interbody fusion for treatment of failed back surgery syndrome: an outcome analysis. Neurosurgery 2004;**54**(3):636-43; discussion 43-4

For peer review only

For peer review only - http://bmjopen.bmj.com/site/about/guidelines.xhtml

Table1.1 Unmatched baseline characteristics and primary outcomes of patients received laminectomy or disectomy surgeries

	Disectomy N=27,867	Laminectomy N=38,887	p-value
Age	47.83±15.58	59.91±14.02	<.0001
Age Group			<.0001
<20	416(1.49)	232(0.60)	
20-39	8987(32.25)	3667(9.43)	
40-59	11511(41.31)	13030(33.51)	
60-79	6663(23.91)	20561(52.87)	
>=80	290(1.04)	1397(3.59)	
Gender			<.0001
Female	10629(38.14)	21335(54.86)	
Male	17238(61.86)	17552(45.14)	
Comorbidities			
Myocardial infarct	149(0.53)	404(1.04)	<.0001
Congestive heart failure	436(1.56)	1632(4.20)	<.0001
Peripheral vascular disease	196(0.70)	630(1.62)	<.0001
Cerebrovascular disease	1320(4.74)	4050(10.41)	<.0001
Dementia	199(0.71)	632(1.63)	<.0001
Chronic lung disease	514(1.84)	1620(4.17)	<.0001
Connective tissue disease	80(0.29)	132(0.34)	0.2357
Ulcer	5528(19.84)	11362(29.22)	<.0001
Chronic liver disease	2593(9.30)	4768(12.26)	<.0001
Diabetes	2291(8.22)	5741(14.76)	<.0001
Diabetes with end organ damage	761(2.73)	2029(5.22)	<.0001
Hemiplegia	80(0.29)	238(0.61)	<.0001
Moderate or severe kidney disease	545(1.96)	1590(4.09)	<.0001
Tumor, leukemia, lymphoma	20(0.07)	49(0.13)	0.0315
Moderate or severe liver disease	52(0.19)	98(0.25)	0.0784
Malignant tumor, metastasis			-
AIDS	4(0.01)	3(0.01)	0.4087
Spinal revision surgery (3 month)	765(2.75)	459(1.18)	<.0001
Disectomy	449(1.61)	128(0.33)	<.0001
Laminectomy	187(0.67)	196(0.5)	0.0048
Spinal fusion	129(0.46)	135(0.35)	0.0188
Spinal revision surgery (3 month~ 1 year)	941(3.38)	999(2.57)	<.0001
Disectomy	389(1.40)	186(0.48)	<.0001
Laminectomy	287(1.03)	406(1.04)	0.8587
Spinal fusion	265(0.95)	407(1.05)	0.2220
Spinal revision surgery (>1 year)	2718(9.75)	3770(9.69)	0.8006
Disectomy	844(3.03)	485(1.25)	<.0001
Laminectomy	708(2.54)	1282(3.3)	<.0001
Spinal fusion	1166(4.18)	2003(5.15)	<.0001
Total spinal revision surgery	4424(15.88)	5228(13.44)	<.0001
Disectomy	1682(6.04)	799(2.05)	<.0001
Laminectomy	1182(4.24)	1884(4.84)	0.0002
Spinal fusion	1560(5.60)	2545(6.54)	<.0001
Final spinal fusion	3136(11.25)	4699(12.08)	0.0010

For peer review only - http://bmjopen.bmj.com/site/about/guidelines.xhtml

For peer review only

Death	3900(14.00)	8545(21.97)	<.0001

For peer review only

For peer review only - http://bmjopen.bmj.com/site/about/guidelines.xhtml

Table1.2 matched baseline characteristics and primary outcomes of patients received laminectomy or disectomy surgeries

	Disectomy N=8,024	Laminectomy N=8,024	p-value
Age	40.16±11.26	40.51±11.51	0.0536
Age Group			0.3398
<20	195(2.43)	217(2.70)	
20-39	3621(45.13)	3500(43.62)	
40-59	3922(48.88)	4023(50.14)	
60-79	246(3.07)	244(3.04)	
>=80	40(0.50)	40(0.50)	
Gender			1.0000
Female	2225(27.73)	2225(27.73)	
Male	5799(72.27)	5799(72.27)	
Comorbidities			
Myocardial infarct	32(0.40)	34(0.42)	0.8051
Congestive heart failure	87(1.08)	88(1.10)	0.9394
Peripheral vascular disease	49(0.61)	60(0.75)	0.2904
Cerebrovascular disease	215(2.68)	220(2.74)	0.8080
Dementia	41(0.51)	44(0.55)	0.7442
Chronic lung disease	86(1.07)	79(0.98)	0.5838
Connective tissue disease	15(0.19)	17(0.21)	0.7234
Ulcer	1124(14.01)	1129(14.07)	0.9095
Chronic liver disease	705(8.79)	693(8.64)	0.7369
Diabetes	431(5.37)	412(5.13)	0.5014
Diabetes with end organ damage	150(1.87)	144(1.79)	0.7240
Hemiplegia	18(0.22)	17(0.21)	0.8656
Moderate or severe kidney disease	107(1.33)	113(1.41)	0.6838
Tumor, leukemia, lymphoma	3(0.04)	4(0.05)	0.7054
Moderate or severe liver disease	7(0.09)	10(0.12)	0.4666
Malignant tumor, metastasis			-
AIDS			-
Spinal revision surgery (3 month)	208(2.59)	123(1.53)	<.0001
Disectomy	128(1.60)	48(0.60)	<.0001
Laminectomy	46(0.57)	37(0.46)	0.3220
Spinal fusion	34(0.42)	38(0.47)	0.6366
Spina revision l surgery (3 month~ 1 year)	241(3.00)	189(2.36)	0.0110
Disectomy	109(1.36)	54(0.67)	<.0001
Laminectomy	58(0.72)	63(0.79)	0.6482
Spinal fusion	74(0.92)	72(0.90)	0.8679
Spinal revision surgery (>1 year)	675(8.41)	665(8.29)	0.7754
Disectomy	278(3.46)	181(2.26)	<.0001
Laminectomy	132(1.65)	164(2.04)	0.0605
Spinal fusion	265(3.30)	320(3.99)	0.0205
Total spinal revision surgery	1124(14.01)	977(12.18)	0.0006
Disectomy	515(6.42)	283(3.53)	<.0001
Laminectomy	236(2.94)	264(3.29)	0.2033
Spinal fusion	373(4.65)	430(5.36)	0.0390
Final spinal fusion	784(9.77)	838(10.44)	0.1573

For peer review only

For peer review only - http://bmjopen.bmj.com/site/about/guidelines.xhtml

Death	795(9.91)	884(11.02)	0.0217

For peer review only

For peer review only - http://bmjopen.bmj.com/site/about/guidelines.xhtml

Table2. Multivariate Cox proportional hazard models for revision lumbar spine surgical rates between disectomy and laminectomy with or without matched data

	Unmatched		Matched	
	sHR(95%CI)	p-value	sHR(95%CI)	p-value
Laminectomy vs. Disectomy	0.81(0.78-0.85)	<.0001	0.86(0.79-0.94)	0.0007
age	1.01(1.00-1.01)	<.0001	1.01(1.00-1.01)	0.0007
Male vs. Female	1.09(1.05-1.14)	<.0001	1.09(0.99-1.20)	0.0937
Comorbidities				
Myocardial infarct	1.15(0.94-1.42)	0.1825	1.21(0.69-2.14)	0.5097
Congestive heart failure	1.04(0.92-1.18)	0.4979	1.30(0.89-1.90)	0.1751
Peripheral vascular disease	0.73(0.59-0.91)	0.0046	0.82(0.48-1.41)	0.4788
Cerebrovascular disease	0.99(0.91-1.07)	0.7413	0.97(0.73-1.28)	0.8136
Dementia	1.11(0.92-1.33)	0.2813	1.19(0.69-2.05)	0.5300
Chronic lung disease	1.05(0.94-1.18)	0.4067	1.00(0.67-1.50)	0.9833
Connective tissue disease	1.16(0.83-1.61)	0.3925	1.73(0.86-3.49)	0.1262
Ulcer	0.96(0.92-1.01)	0.1285	1.12(0.99-1.27)	0.0854
Chronic liver disease	0.99(0.92-1.06)	0.7486	1.14(0.98-1.33)	0.0917
Diabetes	1.09(1.01-1.17)	0.0263	1.14(0.92-1.42)	0.2392
Diabetes with end organ damage	1.12(1.00-1.25)	0.0590	0.99(0.70-1.40)	0.9436
Hemiplegia	1.18(0.88-1.57)	0.2672	1.18(0.52-2.72)	0.6897
Moderate or severe kidney disease	1.09(0.97-1.23)	0.1319	0.83(0.57-1.22)	0.3431
Tumor, leukemia, lymphoma	1.40(0.80-2.47)	0.2434	NA	
Moderate or severe liver disease	1.36(0.90-2.06)	0.1399	1.34(0.43-4.22)	0.6124
Malignant tumor, metastasis	NA		NA	.
AIDS	1.11(0.16-7.90)	0.9149	NA	.

sHR: subdistribution hazard ratio

For peer review only - http://bmjopen.bmj.com/site/about/guidelines.xhtml

Table3. Multivariate Cox proportional hazard models for final revision lumbar spine fusion rates between disectomy and laminectomy with or without matched data

	Unmatched		Matched	
	sHR(95%CI)	p-value	sHR(95%CI)	p-value
Laminectomy vs. Disectomy	1.05(1.00-1.10)	0.0524	1.11(1.01-1.22)	0.0377
age	1.00(1.00-1.01)	<.0001	1.02(1.01-1.02)	<.0001
Comorbidities				
Myocardial infarct	1.16(0.92-1.45)	0.2131	0.95(0.47-1.91)	0.8832
Congestive heart failure	1.06(0.93-1.21)	0.4071	1.16(0.75-1.78)	0.5045
Peripheral vascular disease	0.96(0.78-1.18)	0.6927	0.90(0.52-1.57)	0.7183
Cerebrovascular disease	1.04(0.95-1.13)	0.3858	1.07(0.80-1.45)	0.6419
Dementia	1.13(0.93-1.38)	0.2320	0.87(0.45-1.69)	0.6863
Chronic lung disease	1.15(1.01-1.30)	0.0351	0.95(0.61-1.50)	0.8351
Connective tissue disease	0.89(0.59-1.34)	0.5653	1.09(0.46-2.60)	0.8492
Ulcer	1.18(1.12-1.24)	<.0001	1.34(1.16-1.55)	<.0001
Chronic liver disease	1.21(1.13-1.30)	<.0001	1.37(1.16-1.62)	0.0002
Diabetes	1.29(1.19-1.39)	<.0001	1.19(0.93-1.54)	0.1730
Diabetes with end organ damage	1.11(0.98-1.25)	0.0887	0.95(0.65-1.40)	0.7954
Hemiplegia	1.12(0.80-1.56)	0.5194	0.43(0.10-1.80)	0.2471
Moderate or severe kidney disease	1.20(1.06-1.36)	0.0042	1.04(0.71-1.53)	0.8466
Tumor, leukemia, lymphoma	1.31(0.71-2.41)	0.3819	NA	
Moderate or severe liver disease	1.36(0.87-2.13)	0.1778	1.02(0.26-4.01)	0.9728
Malignant tumor, metastasis	NA		NA	.
AIDS	1.91(0.32-11.39)	0.4762	NA	.

sHR: subdistribution hazard ratio

For peer review only - http://bmjopen.bmj.com/site/about/guidelines.xhtml

FIGURE LEGENDS:

Fig 1. The cumulated incidence of total revision spinal surgery after the first time spinal surgeries

Fig 2. The cumulated incidence of final spinal fusion surgery after the first time spinal surgeries

For peer review only

For peer review only - http://bmjopen.bmj.com/site/about/guidelines.xhtml

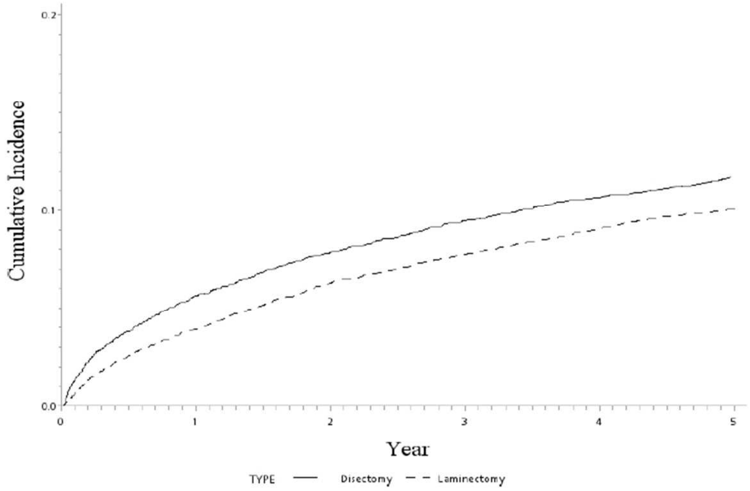

Disectomy	8024	7446	7193	6993	6824	6687
Laminectomy	8024	7578	7281	7088	6901	6737

	1st	2nd	3rd
Disectomy	0.0563(0.0515-0.0616)	0.0784(0.0727-0.0845)	0.0945(0.0882-0.1012)
Laminectomy	0.0392(0.0352-0.0437)	0.0628(0.0577-0.0684)	0.0775(0.0718-0.0836)
	4th	5th	
Disectomy	0.1066(0.1-0.1136)	0.1169(0.1099-0.1242)	
Laminectomy	0.0906(0.0844-0.0971)	0.1006(0.0942-0.1075)	

Fig 1. The cumulated incidence of total revision spinal surgery after the first time spinal surgeries

Fig 1. The cumulated incidence of total revision spinal surgery after the first time spinal surgeries

80x67mm (300 x 300 DPI)

only

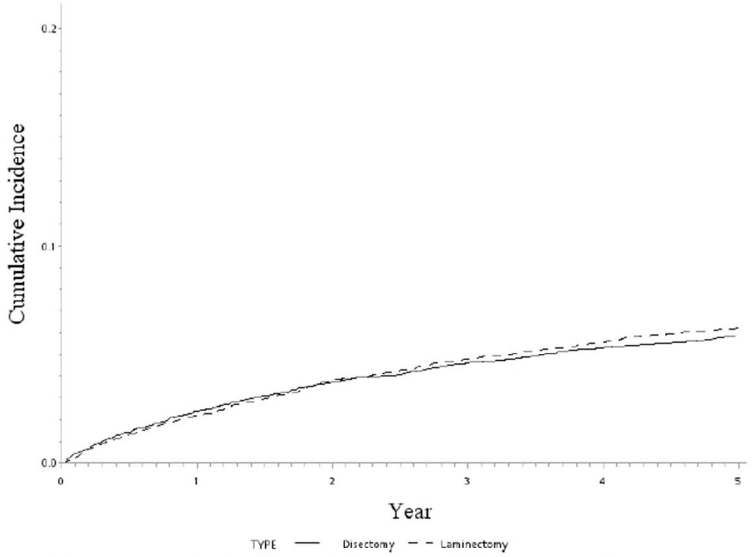

Disectomy	8024	7446	7193	6993	6824	6687
Laminectomy	8024	7578	7281	7088	6901	6737

	1st	2nd	3rd
Disectomy	0.0238(0.0207-0.0275)	0.0371(0.0331-0.0416)	0.0458(0.0413-0.0507)
Laminectomy	0.0216(0.0186-0.0251)	0.0373(0.0333-0.0417)	0.0475(0.043-0.0525)

	4th	5th	
Disectomy	0.0529(0.0481-0.0582)	0.0585(0.0534-0.064)	
Laminectomy	0.0555(0.0506-0.0609)	0.062(0.0568-0.0677)	

Fig 2. The cumulated incidence of final spinal fusion surgery after the first time spinal surgeries

Fig 2. The cumulated incidence of final spinal fusion surgery after the first time spinal surgeries

72x70mm (300 x 300 DPI)

Appendix . ICD-9-CM codes and the corresponding diseases or procedures

Disease or procedures	Corresponding ICD-9-CM codes
Lumbar disectomy	83024C
laminectomy	83002C, 83003C
Spinal fusion	83043B, 83044B, 83045B, 83046B<br>64221B, 64222B, 64224B,64225B, 64226B
Spine fracture	64160B
Ankylosing spondylitis	720; 720.0
Systemic lupus erythematous (SLE)	710.0
Rheumatoid arthritis (RA)	714.XX
Cancers	140.xx-208.xx
Spinal tumor cases	192.2; 192.3; 198.3; 225.3; 225.4; 237.5
Cervical disease	721.x (x =0,1,5,6,7), 722.0, 722.4,<br>722.71, 722.81, 722.91,<br>723.x (x=0 ~ 9)<br>344.xx (xx=00, 01,02,03,04,09)<br>344.1, 344.2, 344.4x (x=0,1,2)<br>805.xx (xx= 00 ~08; 10 ~18)<br>806.xx (xx= 00 ~09; 10 ~19)<br>952.xx (xx=00 ~09)
thoracic disease	721.2, 721.41<br>722.xx (xx=11, 51, 72, 82, 92)<br>724.01<br>805.2, 805.3,<br>806.xx (xx= 20 ~29; 30 ~39),<br>952.xx (xx=10 ~19)
congenital anomaly of spine	756.xx (xx=13,14,15,19),<br>756.4

For peer review only

Tuberculosis of spine (TB)	015.xx (xx= 00~06)
spine infection	711. xx (xx= 08,48,58,68,88,98)
	730.xx (xx= 08,18,28,38,88,98)

Footnotes: ICD-9-CM, International Classification of Diseases, 9th Revision, Clinical Modification;

For peer review only

STROBE Statement—checklist of items that should be included in reports of observational studies

	Item No	Recommendation	Page No
**Title and abstract**	1	(*a*) Indicate the study's design with a commonly used term in the title or the abstract	1
		(*b*) Provide in the abstract an informative and balanced summary of what was done and what was found	2
**Introduction**			
Background/rationale	2	Explain the scientific background and rationale for the investigation being reported	3
Objectives	3	State specific objectives, including any prespecified hypotheses	4
**Methods**			
Study design	4	Present key elements of study design early in the paper	5-6
Setting	5	Describe the setting, locations, and relevant dates, including periods of recruitment, exposure, follow-up, and data collection	5-6
Participants	6	(*a*) *Cohort study*—Give the eligibility criteria, and the sources and methods of selection of participants. Describe methods of follow-up<br>*Case-control study*—Give the eligibility criteria, and the sources and methods of case ascertainment and control selection. Give the rationale for the choice of cases and controls<br>*Cross-sectional study*—Give the eligibility criteria, and the sources and methods of selection of participants	5-6
		(*b*) *Cohort study*—For matched studies, give matching criteria and number of exposed and unexposed<br>*Case-control study*—For matched studies, give matching criteria and the number of controls per case	
Variables	7	Clearly define all outcomes, exposures, predictors, potential confounders, and effect modifiers. Give diagnostic criteria, if applicable	5-6
Data sources/ measurement	8*	For each variable of interest, give sources of data and details of methods of assessment (measurement). Describe comparability of assessment methods if there is more than one group	5
Bias	9	Describe any efforts to address potential sources of bias	7
Study size	10	Explain how the study size was arrived at	5
Quantitative variables	11	Explain how quantitative variables were handled in the analyses. If applicable, describe which groupings were chosen and why	5-6
Statistical methods	12	(*a*) Describe all statistical methods, including those used to control for confounding	7
		(*b*) Describe any methods used to examine subgroups and interactions	
		(*c*) Explain how missing data were addressed	
		(*d*) *Cohort study*—If applicable, explain how loss to follow-up was addressed<br>*Case-control study*—If applicable, explain how matching of cases and controls was addressed<br>*Cross-sectional study*—If applicable, describe analytical methods taking account of sampling strategy	
		(*e*) Describe any sensitivity analyses	

Continued on next page

For peer review only - http://bmjopen.bmj.com/site/about/guidelines.xhtml

**Results**			**Page No**
Participants	13*	(a) Report numbers of individuals at each stage of study—eg numbers potentially eligible, examined for eligibility, confirmed eligible, included in the study, completing follow-up, and analysed	7-8
		(b) Give reasons for non-participation at each stage	
		(c) Consider use of a flow diagram	
Descriptive data	14*	(a) Give characteristics of study participants (eg demographic, clinical, social) and information on exposures and potential confounders	7-8
		(b) Indicate number of participants with missing data for each variable of interest	
		(c) *Cohort study*—Summarise follow-up time (eg, average and total amount)	
Outcome data	15*	*Cohort study*—Report numbers of outcome events or summary measures over time	
		Case-control study—Report numbers in each exposure category, or summary measures of exposure	*7-8*
		Cross-sectional study—Report numbers of outcome events or summary measures	
Main results	16	(*a*) Give unadjusted estimates and, if applicable, confounder-adjusted estimates and their precision (eg, 95% confidence interval). Make clear which confounders were adjusted for and why they were included	9-10
		(*b*) Report category boundaries when continuous variables were categorized	
		(*c*) If relevant, consider translating estimates of relative risk into absolute risk for a meaningful time period	
Other analyses	17	Report other analyses done—eg analyses of subgroups and interactions, and sensitivity analyses	
**Discussion**			
Key results	18	Summarise key results with reference to study objectives	10-12
Limitations	19	Discuss limitations of the study, taking into account sources of potential bias or imprecision. Discuss both direction and magnitude of any potential bias	12-13
Interpretation	20	Give a cautious overall interpretation of results considering objectives, limitations, multiplicity of analyses, results from similar studies, and other relevant evidence	10-12
Generalisability	21	Discuss the generalisability (external validity) of the study results	10-12
**Other information**			
Funding	22	Give the source of funding and the role of the funders for the present study and, if applicable, for the original study on which the present article is based	14

*Give information separately for cases and controls in case-control studies and, if applicable, for exposed and unexposed groups in cohort and cross-sectional studies.

Note: An Explanation and Elaboration article discusses each checklist item and gives methodological background and published examples of transparent reporting. The STROBE checklist is best used in conjunction with this article (freely available on the Web sites of PLoS Medicine at http://www.plosmedicine.org/, Annals of Internal Medicine at http://www.annals.org/, and Epidemiology at http://www.epidem.com/). Information on the STROBE Initiative is available at www.strobe-statement.org.

For peer review only

BMJ Open

Short-term and Long-term Revision Rates after Lumbar Spine Discectomy versus Laminectomy: A Population-Based Cohort Study

Journal:	*BMJ Open*
Manuscript ID	bmjopen-2017-021028.R1
Article Type:	Research
Date Submitted by the Author:	03-May-2018
Complete List of Authors:	kao, Feng-Chen; E-Da Hospital<br>Hsu, Yao-chun; Fu-Jen Catholic University Hospital, Gastroenterology<br>Wang, Chang-Bi ; China Medical University, Clinical Medicine<br>Tu, Yuan-Kun; E-Da Hospital, Orthopedics<br>Liu, Pao-Hsin ; I-Shou University College of Medicine, Biomedical Engineering
**Primary Subject Heading**:	Surgery
Secondary Subject Heading:	Surgery
Keywords:	disectomy, laminectomy, reoperation, revision rate

SCHOLARONE™ Manuscripts

Short-term and Long-term Revision Rates after Lumbar Spine Discectomy versus Laminectomy: A Population-Based Cohort Study

Feng-Chen Kao[1,2], MD, PhD; Yao-Chun Hsu[3-6], MD, PhD; Chang-Bi Wang PhD[7], Yuan-Kun Tu[1,2], MD, PhD; Pao-Hsin Liu [8]PhD

[1]Department of Orthopedics, E-Da Hospital, Kaohsiung, Taiwan;

[2]School of Medicine for International Students, I-Shou University, Kaohsiung, Taiwan;

[3] School of Medicine, [2]Big Data Research Center, Fu-Jen Catholic University, New Taipei, Taiwan;

[4]Division of Gastroenterology, Fu-Jen Catholic University Hospital, New Taipei, Taiwan

[5]Graduate Institute of Clinical Medicine, China Medical University, Taichung, Taiwan

[6]Division of Gastroenterology and Hepatology, E-Da Hospital, Kaohsiung, Taiwan

[7] Graduate Institute of Public Health, China Medical University, Taichung, Taiwan

[8]Department of Biomedical Engineering, I-Shou University, Kaohsiung, Taiwan

Correspondence to ***Yao-Chun Hsu***

Dr. ***Yao-Chun Hsu***, MD, PhD; Department of Division of Gastroenterology, Fu-Jen Catholic University Hospital; No.69, Guizi Rd., Taishan Dist., New Taipei City 24352, Taiwan Tel: +886-7-615-1100; ext. 5903; Fax: +886-7-6150940;

Email: holdenhsu@ntu.edu.tw

ABSTRACT

For peer review only - http://bmjopen.bmj.com/site/about/guidelines.xhtml

Background/Objective: Degenerative diseases of the lumbar spine were managed with discectomy or laminectomy. This study aimed to compare these two surgical treatments in the postoperative revision rates.

Design: A population-based cohort study from analysis of a healthcare database.

Setting: Data were gathered from the Taiwan National Health Insurance Research Database (NHIRD).

Participants: We enrolled 16,048 patients (4,450 women and 11,598 men) with a mean age of 40.34 years who underwent lumbar discectomy or laminectomy for the first time between January 1, 1997, and December 31, 2007. All patients were followed up for 5 years or until death.

Results: Revision rate within 3 months of the index surgery was significantly higher in patients who underwent discectomy (2.75%) than in those who underwent laminectomy (1.18%; $P < 0.0001$). This difference persisted over the first year following the index surgery (3.38% vs. 2.57%). One year afterwards, the revision rates were similar between the discectomy (9.75%) and laminectomy (9.69%) groups. The final spinal fusion surgery rates were also similar between the groups (11.25% vs. 12.08%).

Conclusion: The revision rate after lumbar discectomy was higher than that after laminectomy within 1 year of the index surgery. However, differences were not identified between patient groups for the two procedures with respect to long-term revision rates and the proportion of patients who required final spinal fusion surgery.

For peer review only - http://bmjopen.bmj.com/site/about/guidelines.xhtml

50 **Strengths and limitations of this study:**

52 1. This population-based cohort study encompassed all residents of Taiwan.
53 2. The universal and compulsory national health insurance mitigated attrition bias as no patients were lost to follow-up.
55 3. However, radiographic and pathological data were unavailable in the NHIRD. Therefore, we could not ascertain the level and pathology of the treated spine.
57 4. The physical conditions of the patients could not be evaluated and unmeasured confounding was possible.

59 **Keywords:** discectomy, laminectomy, reoperation, revision rate

For peer review only

For peer review only - http://bmjopen.bmj.com/site/about/guidelines.xhtml

INTRODUCTION

The natural progression of a degenerative spine leads to primary disc herniation and lumbar spinal stenosis, and most patients with these conditions are treated through surgical interventions [1 2]. Lumbar disc herniation is a common manifestation of degenerative lumbar disc disease [3-5] that occurs early in the degenerative cascade and involves tensile failure of the annulus to contain the gel-like nuclear portion of the disc. Although treatment for lumbar herniated discs can be challenging, nonsurgical treatment is effective in most cases [6 7]. However, studies have indicated that surgery provides superior results to nonsurgical treatments, especially with respect to short-term pain relief [3 8].

Lumbar spinal stenosis is a progressive and dynamic disease that constitutes a continuum of pathological changes in the spinal column as a person ages. The likelihood of lumbar spinal stenosis increases during the fifth decade of life and ranges from 1.7% to 8% in the general population [9]. Surgical treatment focuses on a patient's pathological anatomy and involves relieving neurologic compression; surgical procedures are usually more complex than those performed to relieve simple compression [10].

Revision surgery, which is required in many cases of spinal disease after initial surgical treatment [11 12], presents a challenge for spinal surgeons. Surgeons should be attuned to the clinical circumstances that are appropriate for additional surgery and should be technically qualified to address the anatomic and pathologic obstacles involved in repeat surgery. Incidence of revision surgery after lumbar surgical discectomy varies from 0% to approximately 15% [1]. Frymoyer [13] reported incidence of postdiscectomy instability requiring further spinal fusion surgery as high

For peer review only - http://bmjopen.bmj.com/site/about/guidelines.xhtml

as 6.5%. Relatively few reports have specifically addressed revision surgery for lumbar spinal stenosis. Malter and colleagues [12] reported that the 5-year reoperation rate for patients with spinal stenosis was as high as 12%.

To investigate whether spinal reoperation rates differ after lumbar discectomy and laminectomy for lumbar spinal stenosis, we performed a population-based retrospective study of patients' 5-year follow-up data retrieved from the Taiwan National Health Insurance Research Database (NHIRD).

For peer review only

For peer review only - http://bmjopen.bmj.com/site/about/guidelines.xhtml

DATA SHARING STATEMENT

We examined data from the Taiwan NHIRD, which is released by the Taiwan National Health Research Institute (NHRI) for public use. The NHRI covers the medical claims of 22.9 million residents of Taiwan, accounting for >99% of the total population. The NHIRD contains claims data from 1997 to 2013. The Department of Health and the National Health Insurance (NHI) Bureau of Taiwan ensure the completeness and accuracy of the NHIRD. This study was exempt from an ethics review because the medical records released by the insurance authority are encrypted secondary data and have been approved for use in research.

This retrospective population-based cohort study examined data from the Longitudinal NHIRD. Until the end of 2013, all sampled individuals were followed up for outcome identification by using International Classification of Diseases, Ninth Revision, Clinical Modification (ICD-9-CM) codes. This study was approved by the Institutional Review Board of E-Da hospital (EMRP-104-04) and the Taiwan NHRI (NHIRD-103-116). After the application approved by the Taiwan National Health Research Institutes, the data could be used with 5 years limitation. We use all of the available data without any additional unpublished data. This study was exempt from a full review by the Institutional Review Board of E-Da Hospital.

MATERIALS AND METHODS

Patient and Public Involvement

Our study cohort included patients from the NHIRD who underwent lumbar discectomy or laminectomy for the first time between January 1, 1997, and December

For peer review only

For peer review only - http://bmjopen.bmj.com/site/about/guidelines.xhtml

31, 2007. Those who received their first lumbar discectomy or laminectomy after 2007 were excluded because dynamic stabilization systems such as the Wallis system [14] were marketed in Taiwan after 2007. We also excluded individuals who were continually exposed to oral or injected forms of systemic corticosteroids for 6 months or longer, as well as those with diseases such as ankylosing spondylitis, systemic lupus erythematous, rheumatoid arthritis, malignant cancers, spinal tumors, congenital spinal anomalies, spinal tuberculosis, spinal infections, spinal fractures, cervical spinal disease, and thoracic spinal disease; the corresponding ICD-9-CM codes are listed in Appendix 1.

We divided the study cohort into discectomy and laminectomy groups. Each patient's date of discharge from the hospital after their first lumbar discectomy or laminectomy was considered their index date. Revision lumbar spine surgery was defined as a second lumbar spine operation performed after the index date and comprised the following types: lumbar spine discectomy, lumbar spine laminectomy (including laminotomy), and lumbar spinal fusion surgery (with or without instrumentation). The revision rates in the two surgical groups were evaluated and compared, and the groups were propensity-score matched at a ratio of 1:1 based on the baseline characteristics of the patients. We assessed unmatched and matched data in this study.

Comorbidities existing prior to the index date were classified based on Charlson comorbidity index scores [15], and incidences of mortality after index dates were calculated for both groups. Mortality rates were considered when comparing revision rates to eliminate the influence of death on the calculated likelihood of revision surgery. We also calculated and compared the rates of final revision spinal fusion surgery in the two groups. All patients were followed up until death, withdrawal from

For peer review only - http://bmjopen.bmj.com/site/about/guidelines.xhtml

the NHI program, or December 31, 2012.

Statistical Analysis

We use Pearson's chi-square test and Yates's continuity correction to compare qualitative data, whereas the Student's t test was employed for quantitative data. The annual revision rates were calculated with 95% confidence intervals (CIs). The association between revision lumbar spine surgery between discectomy and laminectomy was explored by the Cox proportional hazard model that took into account age, gender, and baseline comorbidity. Our study analyzed the lumbar spine revision surgery rate by using the Fine and Gray regression model to calculate subdistribution hazards, and P values were determined using Gray's test. The subdistribution hazard ratio (sHR) was defined as significant when $P < 0.05$. All statistical tests and calculations were performed using Statistical Analysis Software, Version 9.4 (SAS Institute, Cary, NC, USA).

For peer review only

For peer review only - http://bmjopen.bmj.com/site/about/guidelines.xhtml

RESULTS

Baseline Characteristics of the Patients

Our study cohort consisted of 66,754 patients (31,964 women and 34,790 men). The discectomy group comprised 27,867 patients and the laminectomy group comprised 38,887 patients. The unmatched and matched baseline characteristics and comorbidities of all patients are listed in Tables 1.1 and 1.2. After propensity-score matching, a total of 8024 patients were enrolled in this study. Lumbar spine revision surgery was defined as any of the following types of lumbar surgery performed after initial lumbar surgery: lumbar spine discectomy, lumbar spine laminectomy (including laminotomy), and lumbar spinal fusion surgery (with or without instrumentation). Final spinal fusion surgery referred to lumbar spinal fusion surgery (with or without instrumentation) performed during the follow-up period.

Reasons of Lumbar Spine Revision Surgery

Those causes of lumbar spine revision surgeries are listed in Tables S1.1 and S1.2. The prevalence of incidental durotomy was 0.04%. The proportions of postoperative hemorrhage and postoperative spine infection were 0.18% and 1.73%, respectively. Finally, the lumbar disc pathology rate was 40.74%.

Total Spinal Surgery Revision Rates

The annual revision rates in the discectomy and laminectomy groups were 5.63% (95% CI, 5.15%–6.16%) and 3.92% (95% CI, 3.52%–4.37%), respectively. Values representing cumulative incidence of revision spinal surgery are displayed in Fig. 1. Significant differences in total revision spinal surgery rates between patients

For peer review only - http://bmjopen.bmj.com/site/about/guidelines.xhtml

who received lumbar discectomy and those who received lumbar laminectomy as initial surgery were identified. In the unmatched data, the revision spinal surgery rates in the discectomy and laminectomy groups were 15.88% and 13.44%, respectively ($P < 0.0001$). In the matched data, the corresponding rates were 14.01% and 12.18%, respectively ($P < 0.001$).

Rates for Revision Surgery Performed within 3 Months of Initial Spinal Surgery

The rates for revision spinal surgery performed within 3 months of initial spinal surgery significantly differed between the two groups ($P < 0.0001$). Based on the unmatched data, the revision spinal surgery rates in the discectomy and laminectomy groups were 2.75% and 1.18%, respectively. In the matched data, the corresponding rates were 2.59% and 1.53%, respectively.

Rates for Revision Surgery Performed between 3 Months and 1 Year after Initial Spinal Surgery

The rates for revision spinal surgery performed between 3 months and 1 year after initial spinal surgery also significantly differed between patients who initially received lumbar discectomy and those who initially received lumbar laminectomy. In the unmatched data, the revision spinal surgery rates in the discectomy and laminectomy groups were 3.38% and 2.57%, respectively ($P < 0.0001$). In the matched data, the corresponding rates were 3.00% and 2.36%, respectively ($P < 0.05$).

Rates for Revision Surgery Performed More Than 1 Year after Initial Spinal Surgery

The rates for revision spinal surgery performed more than 1 year after initial spinal

For peer review only - http://bmjopen.bmj.com/site/about/guidelines.xhtml

surgery did not significantly differ between patients who initially received lumbar discectomy and those who initially received lumbar laminectomy. In the unmatched data, the revision spinal surgery rates in the discectomy and laminectomy groups were 9.75% and 9.69%, respectively. In the matched data, the corresponding rates were 8.41% and 8.29%, respectively.

Differences in Multivariate-Adjusted Total Revision Spinal Surgery Rates between Discectomy and Laminectomy Groups

A multivariate-adjusted Cox proportional hazards model revealed independent differences in the unmatched and matched data (adjusted sHRs, 0.81 and 0.86, respectively; 95% CIs, 0.78–0.85 and 0.79–0.94, respectively; Table 2) between the discectomy and laminectomy groups. Analysis of the unmatched data (Table 2) revealed that age (sHR, 1.01; 95% CI, 1.00–1.01), sex (sHR, 1.09; 95% CI, 1.05–1.14), peripheral vascular disease (sHR, 0.73; 95% CI, 0.59–0.91), and diabetes mellitus (DM; sHR, 1.09; 95% CI, 1.01–1.17) were the risk factors responsible for differences in spinal revision rates between the discectomy and laminectomy groups. Analysis of the matched data indicated that age (sHR, 1.01; 95% CI, 1.00–1.01) was the risk factor responsible for differences in spinal revision rates between the two groups.

Rates for Final Spinal Fusion Surgery Performed after Initial Spinal Surgery

The annual revision rates in the discectomy and laminectomy groups were 2.38% (95% CI, 2.07%–2.75%) and 2.16% (95% CI, 1.86%–2.51%), respectively. The value representing cumulative incidence of final spinal fusion surgery performed after initial spinal surgery is displayed in Fig. 2. No significant differences in the rates for final

For peer review only - http://bmjopen.bmj.com/site/about/guidelines.xhtml

spinal fusion surgery performed after initial surgery were identified between patients who initially received lumbar discectomy and those who initially received lumbar laminectomy. In the unmatched data, the final spinal fusion surgery rates in the discectomy and laminectomy groups were 11.25% and 12.08%, respectively. In the matched data, the corresponding rates were 9.77% and 10.44%, respectively.

Differences in Multivariate-Adjusted Rates of Final Spinal Fusion Surgery Performed after Initial Spinal Surgery between Discectomy and Laminectomy Groups

The multivariate-adjusted Cox proportional hazards model revealed no differences in the unmatched data between the discectomy and laminectomy groups (adjusted sHR, 1.05; 95% CI, 1.00–1.10; Table 3). However, the model revealed independent differences in the matched data between the groups (adjusted sHR, 1.11; 95% CI, 1.01–1.22). In the unmatched data analysis (Table 3), age (sHR, 1.00; 95% CI, 1.00–1.01), chronic lung disease (sHR, 1.15; 95% CI, 1.01–1.30), ulcer (sHR, 1.18; 95% CI, 1.12–1.24), chronic liver disease (sHR, 1.21; 95% CI, 1.13–1.30), DM (sHR, 1.29; 95% CI, 1.19–1.39), and moderate or severe kidney disease (sHR, 1.20; 95% CI, 1.06–1.36) were the risk factors for different final spinal fusion rates between the discectomy and laminectomy groups. In the matched data analysis, age (sHR, 1.02; 95% CI, 1.01–1.02), ulcer (sHR, 1.34; 95% CI, 1.16–1.55), and chronic liver disease (sHR, 1.37; 95% CI, 1.16–1.62) were the corresponding risk factors.

For peer review only - http://bmjopen.bmj.com/site/about/guidelines.xhtml

DISCUSSION

Lumbar disc herniation is one of the most common lumbar spine disorders [16]. In 1934, Mixter and Barr [17] identified a link between sciatica and lumbar disc herniation; since this discovery, discectomy through limited laminotomy has been the most common form of surgical management for lumbar disc prolapse in cases of conservative management failure [18]. The efficacy of lumbar discectomy for treating lumbar disc herniation has been demonstrated [19 20]; however, unsatisfactory outcomes after lumbar discectomy have been reported in 5%–20% of cases [21-24]. The Spine Patient Outcomes Research Trial reported that in patients with lumbar disc herniation, the proportions of reoperation within 4 and 8 years of index procedures were as high as 9% for discectomy patients and 13% for laminectomy patients [19]. The most common cause of ongoing disability after lumbar discectomy is recurrent lumbar disc herniation, which occurs in 5%–15% of patients (this incidence proportion increases over time) [21 23 25-28]. In our study cohort, the rates for revision spinal surgery performed within 3 months and 1 year of lumbar discectomy were 2.75% and 3.38%, respectively; those for revision surgery performed after 1 year and of total revision surgery were 9.75% and 15.88%, respectively.

Lumbar stenosis is caused by spondylotic changes in the facet joints, spinal instability, or a congenitally small spinal canal [29]. Laminectomy remains the standard treatment for spinal stenosis when the spine does not exhibit instability [29]. Despite adequate lumbar decompression, substantial postoperative back and leg pain occur in 10%–15% of patients [30]. Historically, a high proportion of lumbar laminectomies fail, and the proportion of patients who experience recurrent back pain may reach 47% [31 32]. No reoperation rates after lumbar laminectomy without spinal fusion surgery have been reported. In our study, the rates for revision spinal surgery

For peer review only - http://bmjopen.bmj.com/site/about/guidelines.xhtml

performed within 3 months and 1 year of lumbar laminectomy were 1.18% and 2.57%, respectively; those for revision surgery performed after 1 year and for total revision surgery were 9.69% and 13.44%, respectively.

Spinal structures that contribute to spinal stability in certain proportions of patients are as follows: facet capsule, 39%; disc and annulus, 29%; supraspinous and intraspinous ligaments, 19%; and ligamentum flavum, 13% [33]. Interventions at the hemilamina and ligamentum flavum can change both the load-bearing and kinematic characteristics of the spine and lead to spinal segment hypermobility and accelerated bone degeneration [34 35]. Even microdiscectomy can increase the risk of single-level instability [36]. Extensive laminectomy can also potentiate spinal instability [37 38]. Lai [39] reported that sacrificing supraspinous ligaments or tendon insertion points in spinous processes can accelerate development of adjacent instability. Incidences of adjacent instability increase with the number of destructed laminae, and far more posterior spinal complexes are destructed in lumbar laminectomy than in lumbar discectomy. Hence, theoretically, lumbar laminectomy causes greater spinal instability than does lumbar discectomy, leading to a higher reoperation rate after lumbar laminectomy.

In contrast to the theoretically expected outcomes, our study revealed independent differences in reoperation rates based on the unmatched and matched data (adjusted sHR, 0.81 and 0.86; 95% CI, 0.78–0.85 and 0.79–0.94, respectively) between the discectomy and laminectomy groups. Based on the unmatched data, revision spinal surgery rates in the discectomy and laminectomy groups were 15.88% and 13.44%, respectively ($P < 0.0001$). According to the matched data, the corresponding rates in the discectomy and laminectomy groups were 14.01% and 12.18%, respectively ($P < 0.001$). Compared with the laminectomy group, the discectomy group had higher rates

For peer review only - http://bmjopen.bmj.com/site/about/guidelines.xhtml

of reoperation within 3 months and between 3 months and 1 year after initial surgery ($P < 0.05$). However, beyond 1 year, the reoperation rates did not significantly differ between the laminectomy and discectomy groups.

Numerous reasons for reoperation after discectomy have been suggested. Early recurrence may be due to reherniation, infection, or arachnoiditis, whereas late recurrence may be attributed to foraminal stenosis, a painful disc, epidural fibrosis, iatrogenic segmental instability, progressive facet degeneration, or sacroiliac joint pain [40-42]. Outcomes based on natural degeneration of the lumbar spine more than 1 year after initial lumbar spine surgery were similar in the discectomy and laminectomy groups.

North et al. [43] reported that incidence of instability increased from 12.5% after initial revision surgery to 50% after the fourth surgery. Fusion of the symptomatic spinal segment during revision spinal surgery is related to successful outcomes [44-47]. In our study, no significant differences were observed in the final spinal fusion surgery rates after initial spinal surgery between patients who received lumbar discectomy (11.25%) and those who received lumbar laminectomy (12.08%).

Our study had some limitations. First, the laboratory, radiographic, and pathological data of the patients were unavailable in the NHIRD. Thus, we were unable to differentiate between true lumbar disc prolapse and spinal canal stenosis. Second, the physical conditions of the study cohort patients could not be evaluated; this may have led to healthy patient bias. Nevertheless, this stringent definition would have biased the results toward a null association rather than creating a spurious one. In addition, the potential influence of body weight, habitual cigarette smoking, alcohol consumption, and dietary habits could not be assessed because related information was unavailable in the NHIRD. We were also unable to acquire direct information on

For peer review only - http://bmjopen.bmj.com/site/about/guidelines.xhtml

these factors because linking the NHIRD with external databases is strictly prohibited for privacy protection. However, an advantage of the NHIRD is its inclusion of information on 99% of the residents of Taiwan, and no patients in our NHIRD study cohort were lost to follow-up. The complete follow-up in this study was particularly attributable to hospital accessibility.

In conclusion, rates for reoperation within 1 year were higher after lumbar discectomy than after lumbar laminectomy. Beyond 1 year after initial lumbar surgery, reoperation rates and final lumbar spinal fusion surgery rates were similar in the discectomy and laminectomy groups.

For peer review only

ACKNOWLEDGMENTS

This study used data from the National Health Insurance Research Database, which is released by the Bureau of National Health Insurance, Department of Health, and managed by the National Health Research Institutes. The interpretation and conclusions contained herein do not represent the views of the Bureau of National Health Insurance, Department of Health, or National Health Research Institutes. The authors are grateful to Ms. Tzu-Shan Chen for her helpful assistance.

A. Contribution statement

- Substantial contributions to the conception or design of this work or the acquisition, analysis, or interpretation of data for this work: Hsu and Tu

For peer review only - http://bmjopen.bmj.com/site/about/guidelines.xhtml

- Drafting the work or revising it critically for important intellectual content: Kao, Wang, and Liu
- Final approval of the version to be published: Wang and Liu
- Agreement to be accountable for all aspects of this work in ensuring that questions related to the accuracy or integrity of any part of this work are appropriately investigated and resolved: Hsu and Tu

B. Competing interests: This study is being considered for publication only in BMJ Open journal.

C. Funding: This work was supported by the Center for Database Research, E-DA HEALTHCARE GROUP, and E-Da Hospital (EDAHP103048).

D. Data sharing statement: This study was based on data from the National Health Insurance Research Database provided by the Bureau of National Health Insurance, Department of Health, and managed by the National Health Research Institutes. The interpretation and conclusions contained herein do not represent those of Bureau of National Health Insurance, Department of Health, or National Health Research Institutes.

For peer review only

REFERENCES

1. Hoffman RM, Wheeler KJ, Deyo RA. Surgery for herniated lumbar discs: a literature synthesis. Journal of general internal medicine 1993;**8**(9):487-96
2. McCulloch JA. Focus issue on lumbar disc herniation: macro- and microdiscectomy. Spine 1996;**21**(24 Suppl):45s-56s
3. Atlas SJ, Keller RB, Chang Y, et al. Surgical and nonsurgical management of sciatica secondary to a lumbar disc herniation: five-year outcomes from the Maine Lumbar Spine Study. Spine 2001;**26**(10):1179-87
4. DePalma AF, Rothman RH. Surgery of the lumbar spine. Clinical orthopaedics and related research 1969;**63**:162-70
5. Fisher RG, Saunders RL. Lumbar disc protrusion in children. Journal of neurosurgery 1981;**54**(4):480-3 doi: 10.3171/jns.1981.54.4.0480[published Online First: Epub Date]|.
6. Saal JA, Saal JS. Nonoperative treatment of herniated lumbar intervertebral disc with radiculopathy. An outcome study. Spine 1989;**14**(4):431-7

For peer review only - http://bmjopen.bmj.com/site/about/guidelines.xhtml

7. Saal JA, Saal JS, Herzog RJ. The natural history of lumbar intervertebral disc extrusions treated nonoperatively. Spine 1990;**15**(7):683-6
8. Weber H. Lumbar disc herniation. A controlled, prospective study with ten years of observation. Spine 1983;**8**(2):131-40
9. Rosen CD, Kahanovitz N, Bernstein R, et al. A retrospective analysis of the efficacy of epidural steroid injections. Clinical orthopaedics and related research 1988(228):270-2
10. Porter RW. Spinal stenosis and neurogenic claudication. Spine 1996;**21**(17):2046-52
11. Keskimaki I, Seitsalo S, Osterman H, et al. Reoperations after lumbar disc surgery: a population-based study of regional and interspecialty variations. Spine 2000;**25**(12):1500-8
12. Malter AD, McNeney B, Loeser JD, et al. 5-year reoperation rates after different types of lumbar spine surgery. Spine 1998;**23**(7):814-20
13. Frymoyer JW. Back pain and sciatica. The New England journal of medicine 1988;**318**(5):291-300 doi: 10.1056/nejm198802043180506[published Online First: Epub Date]|.
14. Senegas J. Mechanical supplementation by non-rigid fixation in degenerative intervertebral lumbar segments: the Wallis system. European spine journal : official publication of the European Spine Society, the European Spinal Deformity Society, and the European Section of the Cervical Spine Research Society 2002;**11 Suppl 2**:S164-9 doi: 10.1007/s00586-002-0423-9[published Online First: Epub Date]|.
15. Charlson ME, Pompei P, Ales KL, et al. A new method of classifying prognostic comorbidity in longitudinal studies: development and validation. Journal of chronic diseases 1987;**40**(5):373-83
16. Li Z, Tang J, Hou S, et al. Four-year follow-up results of transforaminal lumbar interbody fusion as revision surgery for recurrent lumbar disc herniation after conventional discectomy. Journal of clinical neuroscience : official journal of the Neurosurgical Society of Australasia 2015;**22**(2):331-7 doi: 10.1016/j.jocn.2014.06.098[published Online First: Epub Date]|.
17. MIXTER WJ, BARR JS. Rupture of the Intervertebral Disc with Involvement of the Spinal Canal. New England Journal of Medicine 1934;**211**(5):210-15 doi: 10.1056/nejm193408022110506[published Online First: Epub Date]|.
18. Morgan-Hough CV, Jones PW, Eisenstein SM. Primary and revision lumbar discectomy. A 16-year review from one centre. The Journal of bone and joint surgery British volume 2003;**85**(6):871-4
19. Lurie JD, Tosteson TD, Tosteson AN, et al. Surgical versus nonoperative treatment for lumbar disc herniation: eight-year results for the spine patient outcomes research trial. Spine 2014;**39**(1):3-16 doi: 10.1097/brs.0000000000000088[published Online First: Epub Date]|.
20. Yorimitsu E, Chiba K, Toyama Y, et al. Long-term outcomes of standard discectomy for lumbar disc herniation: a follow-up study of more than 10 years. Spine 2001;**26**(6):652-7
21. Cinotti G, Roysam GS, Eisenstein SM, et al. Ipsilateral recurrent lumbar disc herniation. A prospective, controlled study. The Journal of bone and joint surgery British volume 1998;**80**(5):825-32
22. Cheng J, Wang H, Zheng W, et al. Reoperation after lumbar disc surgery in two hundred and seven patients. International orthopaedics 2013;**37**(8):1511-7 doi: 10.1007/s00264-013-1925-2[published Online First: Epub Date]|.

For peer review only - http://bmjopen.bmj.com/site/about/guidelines.xhtml

23. Aizawa T, Ozawa H, Kusakabe T, et al. Reoperation for recurrent lumbar disc herniation: a study over a 20-year period in a Japanese population. Journal of orthopaedic science : official journal of the Japanese Orthopaedic Association 2012;**17**(2):107-13 doi: 10.1007/s00776-011-0184-6[published Online First: Epub Date]|.
24. Carragee EJ, Han MY, Suen PW, et al. Clinical outcomes after lumbar discectomy for sciatica: the effects of fragment type and anular competence. The Journal of bone and joint surgery American volume 2003;**85-a**(1):102-8
25. Swartz KR, Trost GR. Recurrent lumbar disc herniation. Neurosurgical focus 2003;**15**(3):E10
26. Ruetten S, Komp M, Merk H, et al. Recurrent lumbar disc herniation after conventional discectomy: a prospective, randomized study comparing full-endoscopic interlaminar and transforaminal versus microsurgical revision. Journal of spinal disorders & techniques 2009;**22**(2):122-9 doi: 10.1097/BSD.0b013e318175ddb4[published Online First: Epub Date]|.
27. Ambrossi GL, McGirt MJ, Sciubba DM, et al. Recurrent lumbar disc herniation after single-level lumbar discectomy: incidence and health care cost analysis. Neurosurgery 2009;**65**(3):574-8; discussion 78 doi: 10.1227/01.neu.0000350224.36213.f9[published Online First: Epub Date]|.
28. Miwa S, Yokogawa A, Kobayashi T, et al. Risk factors of recurrent lumbar disk herniation: a single center study and review of the literature. Journal of spinal disorders & techniques 2015;**28**(5):E265-9 doi: 10.1097/BSD.0b013e31828215b3[published Online First: Epub Date]|.
29. Herkowitz H, Garfin, Eismont, Bell & Balderston. *Rothman-Simeone The Spine*. 6 ed, 2011.
30. Booth KC, Bridwell KH, Eisenberg BA, et al. Minimum 5-year results of degenerative spondylolisthesis treated with decompression and instrumented posterior fusion. Spine 1999;**24**(16):1721-7
31. Jackson RK. The long-term effects of wide laminectomy for lumbar disc excision. A review of 130 patients. The Journal of bone and joint surgery British volume 1971;**53**(4):609-16
32. Fritsch EW, Heisel J, Rupp S. The failed back surgery syndrome: reasons, intraoperative findings, and long-term results: a report of 182 operative treatments. Spine 1996;**21**(5):626-33
33. Adams MA, Hutton WC. The mechanical function of the lumbar apophyseal joints. Spine 1983;**8**(3):327-30
34. Kaigle AM, Holm SH, Hansson TH. Experimental instability in the lumbar spine. Spine 1995;**20**(4):421-30
35. Nachemson A. Lumbar spine instability. A critical update and symposium summary. Spine 1985;**10**(3):290-1
36. Schaller B. Failed back surgery syndrome: the role of symptomatic segmental single-level instability after lumbar microdiscectomy. European spine journal : official publication of the European Spine Society, the European Spinal Deformity Society, and the European Section of the Cervical Spine Research Society 2004;**13**(3):193-8 doi: 10.1007/s00586-003-0632-x[published Online First: Epub Date]|.
37. Ebara S, Harada T, Hosono N, et al. Intraoperative measurement of lumbar spinal instability. Spine 1992;**17**(3 Suppl):S44-50
38. Iida Y, Kataoka O, Sho T, et al. Postoperative lumbar spinal instability occurring or progressing secondary to laminectomy. Spine 1990;**15**(11):1186-9

For peer review only - http://bmjopen.bmj.com/site/about/guidelines.xhtml

39. Lai PL, Chen LH, Niu CC, et al. Relation between laminectomy and development of adjacent segment instability after lumbar fusion with pedicle fixation. Spine 2004;**29**(22):2527-32; discussion 32
40. Slipman CW, Shin CH, Patel RK, et al. Etiologies of failed back surgery syndrome. Pain medicine (Malden, Mass) 2002;**3**(3):200-14; discussion 14-7 doi: 10.1046/j.1526-4637.2002.02033.x[published Online First: Epub Date]|.
41. Burton CV, Kirkaldy-Willis WH, Yong-Hing K, et al. Causes of failure of surgery on the lumbar spine. Clinical orthopaedics and related research 1981(157):191-9
42. Waguespack A, Schofferman J, Slosar P, et al. Etiology of long-term failures of lumbar spine surgery. Pain medicine (Malden, Mass) 2002;**3**(1):18-22 doi: 10.1046/j.1526-4637.2002.02007.x[published Online First: Epub Date]|.
43. North RB, Campbell JN, James CS, et al. Failed back surgery syndrome: 5-year follow-up in 102 patients undergoing repeated operation. Neurosurgery 1991;**28**(5):685-90; discussion 90-1
44. Lakkol S, Bhatia C, Taranu R, et al. Efficacy of less invasive posterior lumbar interbody fusion as revision surgery for patients with recurrent symptoms after discectomy. The Journal of bone and joint surgery British volume 2011;**93**(11):1518-23 doi: 10.1302/0301-620x.93b11.27187[published Online First: Epub Date]|.
45. Wong CB, Chen WJ, Chen LH, et al. Clinical outcomes of revision lumbar spinal surgery: 124 patients with a minimum of two years of follow-up. Chang Gung medical journal 2002;**25**(3):175-82
46. Laus M, Alfonso C, Tigani D, et al. Failed back syndrome: a study on 95 patients submitted to reintervention after lumbar nerve root decompression for the treatment of spondylotic lesions. La Chirurgia degli organi di movimento 1994;**79**(1):119-26
47. Duggal N, Mendiondo I, Pares HR, et al. Anterior lumbar interbody fusion for treatment of failed back surgery syndrome: an outcome analysis. Neurosurgery 2004;**54**(3):636-43; discussion 43-4

For peer review only

For peer review only - http://bmjopen.bmj.com/site/about/guidelines.xhtml

FIGURE LEGENDS:

Fig 1. The cumulated incidence of total revision spinal surgery after the first time spinal surgeries

Fig 2. The cumulated incidence of final spinal fusion surgery after the first time spinal surgeries

For peer review only

For peer review only - http://bmjopen.bmj.com/site/about/guidelines.xhtml

Table1.1 Unmatched baseline characteristics and primary outcomes of patients received laminectomy or discectomy surgeries

	Discectomy N=27,867	Laminectomy N=38,887	p-value
Age	47.83±15.58	59.91±14.02	<.0001
Age Group			<.0001
<20	416(1.49)	232(0.60)	
20-39	8987(32.25)	3667(9.43)	
40-59	11511(41.31)	13030(33.51)	
60-79	6663(23.91)	20561(52.87)	
>=80	290(1.04)	1397(3.59)	
Gender			<.0001
Female	10629(38.14)	21335(54.86)	
Male	17238(61.86)	17552(45.14)	
Comorbidities			
Myocardial infarct	149(0.53)	404(1.04)	<.0001
Congestive heart failure	436(1.56)	1632(4.20)	<.0001
Peripheral vascular disease	196(0.70)	630(1.62)	<.0001
Cerebrovascular disease	1320(4.74)	4050(10.41)	<.0001
Dementia	199(0.71)	632(1.63)	<.0001
Chronic lung disease	514(1.84)	1620(4.17)	<.0001
Connective tissue disease	80(0.29)	132(0.34)	0.2357
Ulcer	5528(19.84)	11362(29.22)	<.0001
Chronic liver disease	2593(9.30)	4768(12.26)	<.0001
Diabetes	2291(8.22)	5741(14.76)	<.0001
Diabetes with end organ damage	761(2.73)	2029(5.22)	<.0001
Hemiplegia	80(0.29)	238(0.61)	<.0001
Moderate or severe kidney disease	545(1.96)	1590(4.09)	<.0001
Tumor, leukemia, lymphoma	20(0.07)	49(0.13)	0.0315
Moderate or severe liver disease	52(0.19)	98(0.25)	0.0784
Malignant tumor, metastasis			-
AIDS	4(0.01)	3(0.01)	0.4087
Spinal revision surgery (3 month)	765(2.75)	459(1.18)	<.0001
Discectomy	449(1.61)	128(0.33)	<.0001
Laminectomy	187(0.67)	196(0.5)	0.0048
Spinal instrumentation	129(0.46)	135(0.35)	0.0188
Spinal revision surgery (3 month~ 1 year)	941(3.38)	999(2.57)	<.0001
Discectomy	389(1.40)	186(0.48)	<.0001
Laminectomy	287(1.03)	406(1.04)	0.8587
Spinal instrumentation	265(0.95)	407(1.05)	0.2220
Spinal revision surgery (>1 year)	2718(9.75)	3770(9.69)	0.8006
Discectomy	844(3.03)	485(1.25)	<.0001
Laminectomy	708(2.54)	1282(3.3)	<.0001
Spinal instrumentation	1166(4.18)	2003(5.15)	<.0001
Total spinal revision surgery	4424(15.88)	5228(13.44)	<.0001
Discectomy	1682(6.04)	799(2.05)	<.0001
Laminectomy	1182(4.24)	1884(4.84)	0.0002

For peer review only

Spinal instrumentation	1560(5.60)	2545(6.54)	<.0001
Final spinal fusion	3136(11.25)	4699(12.08)	0.0010
Death	3900(14.00)	8545(21.97)	<.0001

For peer review only

For peer review only - http://bmjopen.bmj.com/site/about/guidelines.xhtml

Table1.2 matched baseline characteristics and primary outcomes of patients received laminectomy or discectomy surgeries

	Discectomy N=8,024	Laminectomy N=8,024	p-value
Age	40.16±11.26	40.51±11.51	0.0536
Age Group			0.3398
<20	195(2.43)	217(2.70)	
20-39	3621(45.13)	3500(43.62)	
40-59	3922(48.88)	4023(50.14)	
60-79	246(3.07)	244(3.04)	
>=80	40(0.50)	40(0.50)	
Gender			1.0000
Female	2225(27.73)	2225(27.73)	
Male	5799(72.27)	5799(72.27)	
Comorbidities			
Myocardial infarct	32(0.40)	34(0.42)	0.8051
Congestive heart failure	87(1.08)	88(1.10)	0.9394
Peripheral vascular disease	49(0.61)	60(0.75)	0.2904
Cerebrovascular disease	215(2.68)	220(2.74)	0.8080
Dementia	41(0.51)	44(0.55)	0.7442
Chronic lung disease	86(1.07)	79(0.98)	0.5838
Connective tissue disease	15(0.19)	17(0.21)	0.7234
Ulcer	1124(14.01)	1129(14.07)	0.9095
Chronic liver disease	705(8.79)	693(8.64)	0.7369
Diabetes	431(5.37)	412(5.13)	0.5014
Diabetes with end organ damage	150(1.87)	144(1.79)	0.7240
Hemiplegia	18(0.22)	17(0.21)	0.8656
Moderate or severe kidney disease	107(1.33)	113(1.41)	0.6838
Tumor, leukemia, lymphoma	3(0.04)	4(0.05)	0.7054
Moderate or severe liver disease	7(0.09)	10(0.12)	0.4666
Malignant tumor, metastasis			-
AIDS			-
Spinal revision surgery (3 month)	208(2.59)	123(1.53)	<.0001
Discectomy	128(1.60)	48(0.60)	<.0001
Laminectomy	46(0.57)	37(0.46)	0.3220
Spinal instrumentation	34(0.42)	38(0.47)	0.6366
Spinal revision surgery (3 month~ 1 year)	241(3.00)	189(2.36)	0.0110
Discectomy	109(1.36)	54(0.67)	<.0001
Laminectomy	58(0.72)	63(0.79)	0.6482
Spinal instrumentation	74(0.92)	72(0.90)	0.8679
Spinal revision surgery (>1 year)	675(8.41)	665(8.29)	0.7754
Discectomy	278(3.46)	181(2.26)	<.0001
Laminectomy	132(1.65)	164(2.04)	0.0605
Spinal instrumentation	265(3.30)	320(3.99)	0.0205
Total spinal revision surgery	1124(14.01)	977(12.18)	0.0006
Discectomy	515(6.42)	283(3.53)	<.0001
Laminectomy	236(2.94)	264(3.29)	0.2033

For peer review only - http://bmjopen.bmj.com/site/about/guidelines.xhtml

Spinal instrumentation	373(4.65)	430(5.36)	0.0390
Final spinal fusion	784(9.77)	838(10.44)	0.1573
Death	795(9.91)	884(11.02)	0.0217

For peer review only

For peer review only - http://bmjopen.bmj.com/site/about/guidelines.xhtml

Table2. Multivariate Cox proportional hazard models for revision lumbar spine surgical rates between discectomy and laminectomy with or without matched data

	Unmatched		Matched	
	sHR(95%CI)	p-value	sHR(95%CI)	p-value
Laminectomy vs. Discectomy	0.81(0.78-0.85)	<.0001	0.86(0.79-0.94)	0.0007
age	1.01(1.00-1.01)	<.0001	1.01(1.00-1.01)	0.0007
Male vs. Female	1.09(1.05-1.14)	<.0001	1.09(0.99-1.20)	0.0937
Comorbidities				
Myocardial infarct	1.15(0.94-1.42)	0.1825	1.21(0.69-2.14)	0.5097
Congestive heart failure	1.04(0.92-1.18)	0.4979	1.30(0.89-1.90)	0.1751
Peripheral vascular disease	0.73(0.59-0.91)	0.0046	0.82(0.48-1.41)	0.4788
Cerebrovascular disease	0.99(0.91-1.07)	0.7413	0.97(0.73-1.28)	0.8136
Dementia	1.11(0.92-1.33)	0.2813	1.19(0.69-2.05)	0.5300
Chronic lung disease	1.05(0.94-1.18)	0.4067	1.00(0.67-1.50)	0.9833
Connective tissue disease	1.16(0.83-1.61)	0.3925	1.73(0.86-3.49)	0.1262
Ulcer	0.96(0.92-1.01)	0.1285	1.12(0.99-1.27)	0.0854
Chronic liver disease	0.99(0.92-1.06)	0.7486	1.14(0.98-1.33)	0.0917
Diabetes	1.09(1.01-1.17)	0.0263	1.14(0.92-1.42)	0.2392
Diabetes with end organ damage	1.12(1.00-1.25)	0.0590	0.99(0.70-1.40)	0.9436
Hemiplegia	1.18(0.88-1.57)	0.2672	1.18(0.52-2.72)	0.6897
Moderate or severe kidney disease	1.09(0.97-1.23)	0.1319	0.83(0.57-1.22)	0.3431
Tumor, leukemia, lymphoma	1.40(0.80-2.47)	0.2434	NA	
Moderate or severe liver disease	1.36(0.90-2.06)	0.1399	1.34(0.43-4.22)	0.6124
Malignant tumor, metastasis	NA		NA	.
AIDS	1.11(0.16-7.90)	0.9149	NA	.

sHR: subdistribution hazard ratio

For peer review only - http://bmjopen.bmj.com/site/about/guidelines.xhtml

For peer review only

Table3. Multivariate Cox proportional hazard models for final revision lumbar spine fusion rates between discectomy and laminectomy with or without matched data

	Unmatched		Matched	
	sHR(95%CI)	p-value	sHR(95%CI)	p-value
Laminectomy vs. Discectomy	1.05(1.00-1.10)	0.0524	1.11(1.01-1.22)	0.0377
age	1.00(1.00-1.01)	<.0001	1.02(1.01-1.02)	<.0001
Comorbidities				
Myocardial infarct	1.16(0.92-1.45)	0.2131	0.95(0.47-1.91)	0.8832
Congestive heart failure	1.06(0.93-1.21)	0.4071	1.16(0.75-1.78)	0.5045
Peripheral vascular disease	0.96(0.78-1.18)	0.6927	0.90(0.52-1.57)	0.7183
Cerebrovascular disease	1.04(0.95-1.13)	0.3858	1.07(0.80-1.45)	0.6419
Dementia	1.13(0.93-1.38)	0.2320	0.87(0.45-1.69)	0.6863
Chronic lung disease	1.15(1.01-1.30)	0.0351	0.95(0.61-1.50)	0.8351
Connective tissue disease	0.89(0.59-1.34)	0.5653	1.09(0.46-2.60)	0.8492
Ulcer	1.18(1.12-1.24)	<.0001	1.34(1.16-1.55)	<.0001
Chronic liver disease	1.21(1.13-1.30)	<.0001	1.37(1.16-1.62)	0.0002
Diabetes	1.29(1.19-1.39)	<.0001	1.19(0.93-1.54)	0.1730
Diabetes with end organ damage	1.11(0.98-1.25)	0.0887	0.95(0.65-1.40)	0.7954
Hemiplegia	1.12(0.80-1.56)	0.5194	0.43(0.10-1.80)	0.2471
Moderate or severe kidney disease	1.20(1.06-1.36)	0.0042	1.04(0.71-1.53)	0.8466
Tumor, leukemia, lymphoma	1.31(0.71-2.41)	0.3819	NA	
Moderate or severe liver disease	1.36(0.87-2.13)	0.1778	1.02(0.26-4.01)	0.9728
Malignant tumor, metastasis	NA		NA	.
AIDS	1.91(0.32-11.39)	0.4762	NA	.

sHR: subdistribution hazard ratio

For peer review only

For peer review only - http://bmjopen.bmj.com/site/about/guidelines.xhtml

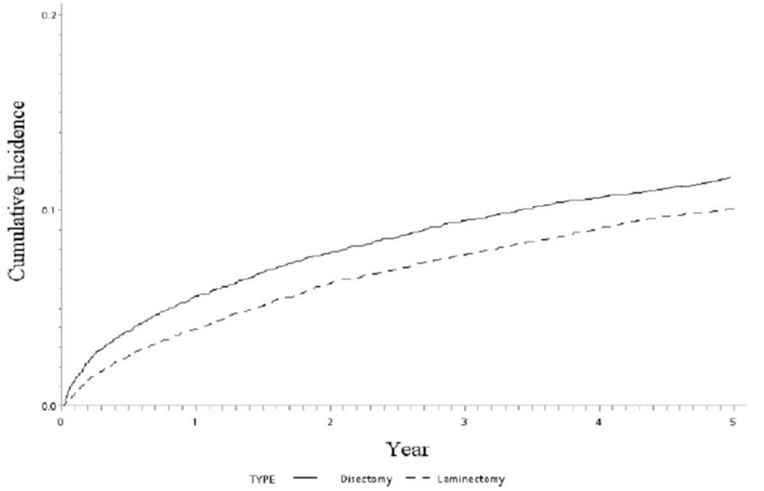

Disectomy	8024	7446	7193	6993	6824	6687
Laminectomy	8024	7578	7281	7088	6901	6737

	1st	2nd	3rd
Disectomy	0.0563(0.0515-0.0616)	0.0784(0.0727-0.0845)	0.0945(0.0882-0.1012)
Laminectomy	0.0392(0.0352-0.0437)	0.0628(0.0577-0.0684)	0.0775(0.0718-0.0836)
	4th	5th	
Disectomy	0.1066(0.1-0.1136)	0.1169(0.1099-0.1242)	
Laminectomy	0.0906(0.0844-0.0971)	0.1006(0.0942-0.1075)	

Fig 1. The cumulated incidence of total revision spinal surgery after the first time spinal surgeries

Fig 1. The cumulated incidence of total revision spinal surgery after the first time spinal surgeries

107x90mm (300 x 300 DPI)

only

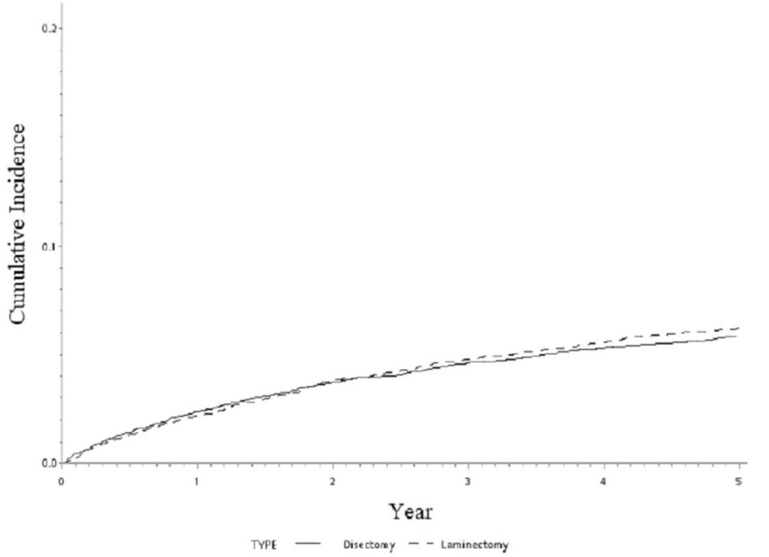

Disectomy	8024	7446	7193	6993	6824	6687
Laminectomy	8024	7578	7281	7088	6901	6737

	1st	2nd	3rd
Disectomy	0.0238(0.0207-0.0275)	0.0371(0.0331-0.0416)	0.0458(0.0413-0.0507)
Laminectomy	0.0216(0.0186-0.0251)	0.0373(0.0333-0.0417)	0.0475(0.043-0.0525)
	4th	5th	
Disectomy	0.0529(0.0481-0.0582)	0.0585(0.0534-0.064)	
Laminectomy	0.0555(0.0506-0.0609)	0.062(0.0568-0.0677)	

Fig 2. The cumulated incidence of final spinal fusion surgery after the first time spinal surgeries

Fig 2. The cumulated incidence of final spinal fusion surgery after the first time spinal surgeries

92x90mm (300 x 300 DPI)

TABLE S1.1 The Cause of revision lumbar spine surgery (Unmatched data)

	Discectomy N=4424	Laminectomy N=5228
Incidental durotomy	3(0.07)	1(0.02)
Post-operative hemorrhage	6(0.14)	11(0.21)
Post-operative spine infection	59(1.33)	108(2.07)
Postlaminectomy syndrome; lumbar region	322(7.28)	543(10.39)
Lumar disc problem	2523(57.03)	1409(26.95)
Acquired spondylolishtesis	386(8.73)	753(14.4)
Lumbar spinal stenosis	605(13.68)	1142(21.84)
Lumbosacral spondylosis	520(11.75)	1261(24.12)

TABLE S1.2 The Cause of revision lumbar spine surgery (matched data)

	Discectomy N=1124	Laminectomy N=977
Incidental durotomy	2(0.18)	1(0.1)
Post-operative hemorrhage	2(0.18)	3(0.31)
Post-operative spine infection	20(1.78)	18(1.84)
Postlaminectomy syndrome; lumbar region	78(6.94)	86(8.8)
Lumar disc problem	763(67.88)	442(45.24)
Acquired spondylolishtesis	53(4.72)	74(7.57)
Lumbar spinal stenosis	93(8.27)	131(13.41)
Lumbosacral spondylosis	113(10.05)	222(22.72)

For peer review only

Appendix . ICD-9-CM codes and the corresponding diseases or procedures

Disease or procedures	Corresponding ICD-9-CM codes
Lumbar discectomy	83024C
laminectomy	83002C, 83003C
Spinal fusion	83043B, 83044B, 83045B, 83046B<br>64221B, 64222B, 64224B,64225B, 64226B
Spine fracture	64160B
Ankylosing spondylitis	720; 720.0
Systemic lupus erythematous (SLE)	710.0
Rheumatoid arthritis (RA)	714.XX
Cancers	140.xx-208.xx
Spinal tumor cases	192.2; 192.3; 198.3; 225.3; 225.4; 237.5
Cervical disease	721.x (x =0,1,5,6,7), 722.0, 722.4,<br>722.71, 722.81, 722.91,<br>723.x (x=0 ~ 9)<br>344.xx (xx=00, 01,02,03,04,09)<br>344.1, 344.2, 344.4x (x=0,1,2)<br>805.xx (xx= 00 ~08; 10 ~18)<br>806.xx (xx= 00 ~09; 10 ~19)<br>952.xx (xx=00 ~09)
thoracic disease	721.2, 721.41<br>722.xx (xx=11, 51, 72, 82, 92)<br>724.01<br>805.2, 805.3,<br>806.xx (xx= 20 ~29; 30 ~39),<br>952.xx (xx=10 ~19)
congenital anomaly of spine	756.xx (xx=13,14,15,19),<br>756.4

For peer review only

Tuberculosis of spine (TB)	015.xx (xx= 00~06)
spine infection	711. xx (xx= 08,48,58,68,88,98)<br>730.xx (xx= 08,18,28,38,88,98)
Incidental durotomy	998.2
Post-operative hemorrhage	998.1x (xx= 1,2,3)
Post-operative spine infection	998.3; 998.6<br>998.xx (xx= 51,59,83)<br>711. xx (xx= 08,48,58,68,88,98)<br>730.xx (xx= 08,18,28,38,88,98)
Postlaminectomy syndrome; lumbar region	722.83; 722.80
Lumar disc problem	722.x (x=2, 6,<br>722.xx (xx=10, 52, 70, 73, 90.93)
Acquired spondylolishtesis	738.4; 738.5
Lumbar spinal stenosis	724.02; 724.09
Lumbosacral spondylosis	721.3;<br>721.xx (xx= 42, 90, 91)<br>722.32; 722.39

Footnotes: ICD-9-CM, International Classification of Diseases, 9th Revision, Clinical Modification;

For peer review only

STROBE Statement—checklist of items that should be included in reports of observational studies

	Item No	Recommendation	Page No
**Title and abstract**	1	(*a*) Indicate the study's design with a commonly used term in the title or the abstract	1
		(*b*) Provide in the abstract an informative and balanced summary of what was done and what was found	2
**Introduction**			
Background/rationale	2	Explain the scientific background and rationale for the investigation being reported	3
Objectives	3	State specific objectives, including any prespecified hypotheses	4
**Methods**			
Study design	4	Present key elements of study design early in the paper	5-6
Setting	5	Describe the setting, locations, and relevant dates, including periods of recruitment, exposure, follow-up, and data collection	5-6
Participants	6	(*a*) *Cohort study*—Give the eligibility criteria, and the sources and methods of selection of participants. Describe methods of follow-up<br>*Case-control study*—Give the eligibility criteria, and the sources and methods of case ascertainment and control selection. Give the rationale for the choice of cases and controls<br>*Cross-sectional study*—Give the eligibility criteria, and the sources and methods of selection of participants	5-6
		(*b*) *Cohort study*—For matched studies, give matching criteria and number of exposed and unexposed<br>*Case-control study*—For matched studies, give matching criteria and the number of controls per case	
Variables	7	Clearly define all outcomes, exposures, predictors, potential confounders, and effect modifiers. Give diagnostic criteria, if applicable	5-6
Data sources/ measurement	8*	For each variable of interest, give sources of data and details of methods of assessment (measurement). Describe comparability of assessment methods if there is more than one group	5
Bias	9	Describe any efforts to address potential sources of bias	7
Study size	10	Explain how the study size was arrived at	5
Quantitative variables	11	Explain how quantitative variables were handled in the analyses. If applicable, describe which groupings were chosen and why	5-6
Statistical methods	12	(*a*) Describe all statistical methods, including those used to control for confounding	7
		(*b*) Describe any methods used to examine subgroups and interactions	
		(*c*) Explain how missing data were addressed	
		(*d*) *Cohort study*—If applicable, explain how loss to follow-up was addressed<br>*Case-control study*—If applicable, explain how matching of cases and controls was addressed<br>*Cross-sectional study*—If applicable, describe analytical methods taking account of sampling strategy	
		(*e*) Describe any sensitivity analyses	

Continued on next page

For peer review only

For peer review only - http://bmjopen.bmj.com/site/about/guidelines.xhtml

Results			Page No
Participants	13*	(a) Report numbers of individuals at each stage of study—eg numbers potentially eligible, examined for eligibility, confirmed eligible, included in the study, completing follow-up, and analysed	7-8
		(b) Give reasons for non-participation at each stage	
		(c) Consider use of a flow diagram	
Descriptive data	14*	(a) Give characteristics of study participants (eg demographic, clinical, social) and information on exposures and potential confounders	7-8
		(b) Indicate number of participants with missing data for each variable of interest	
		(c) *Cohort study*—Summarise follow-up time (eg, average and total amount)	
Outcome data	15*	*Cohort study*—Report numbers of outcome events or summary measures over time	
		Case-control study—Report numbers in each exposure category, or summary measures of exposure	*7-8*
		Cross-sectional study—Report numbers of outcome events or summary measures	
Main results	16	(*a*) Give unadjusted estimates and, if applicable, confounder-adjusted estimates and their precision (eg, 95% confidence interval). Make clear which confounders were adjusted for and why they were included	9-10
		(*b*) Report category boundaries when continuous variables were categorized	
		(*c*) If relevant, consider translating estimates of relative risk into absolute risk for a meaningful time period	
Other analyses	17	Report other analyses done—eg analyses of subgroups and interactions, and sensitivity analyses	
**Discussion**			
Key results	18	Summarise key results with reference to study objectives	10-12
Limitations	19	Discuss limitations of the study, taking into account sources of potential bias or imprecision. Discuss both direction and magnitude of any potential bias	12-13
Interpretation	20	Give a cautious overall interpretation of results considering objectives, limitations, multiplicity of analyses, results from similar studies, and other relevant evidence	10-12
Generalisability	21	Discuss the generalisability (external validity) of the study results	10-12
**Other information**			
Funding	22	Give the source of funding and the role of the funders for the present study and, if applicable, for the original study on which the present article is based	14

*Give information separately for cases and controls in case-control studies and, if applicable, for exposed and unexposed groups in cohort and cross-sectional studies.

Note: An Explanation and Elaboration article discusses each checklist item and gives methodological background and published examples of transparent reporting. The STROBE checklist is best used in conjunction with this article (freely available on the Web sites of PLoS Medicine at http://www.plosmedicine.org/, Annals of Internal Medicine at http://www.annals.org/, and Epidemiology at http://www.epidem.com/). Information on the STROBE Initiative is available at www.strobe-statement.org.

For peer review only
